# The Secretome Analysis of Activated Human Renal Fibroblasts Revealed Beneficial Effect of the Modulation of the Secreted Peptidyl-Prolyl Cis-Trans Isomerase A in Kidney Fibrosis

**DOI:** 10.3390/cells9071724

**Published:** 2020-07-18

**Authors:** Gry H. Dihazi, Marwa Eltoweissy, Olaf Jahn, Björn Tampe, Michael Zeisberg, Hauke S. Wülfrath, Gerhard A. Müller, Hassan Dihazi

**Affiliations:** 1 Institute for Clinical Chemistry/UMG-Laboratories, University Medical Center Göttingen, Robert-Koch-Strasse 40, D-37075 Göttingen, Germany; gryhelene.dihazi@med.uni-goettingen.de (G.H.D.); h.wuelfrath@stud.uni-goettingen.de (H.S.W.); 2 Department of Zoology, Faculty of Science, Alexandria University, Alexandria 21500, Egypt; marwaeltoweissy@alexu.edu.eg; 3 Proteomics Group, Max-Planck-Institute of Experimental Medicine, Hermann-Rein-Strasse 3, D-37075 Göttingen, Germany; jahn@em.mpg.de; 4 Clinic for Nephrology and Rheumatology, University Medical Center Göttingen, Robert-Koch-Strasse 40, D-37075 Göttingen, Germany; bjoern.tampe@med.uni-goettingen.de (B.T.); michael.zeisberg@med.uni-goettingen.de (M.Z.); gmueller@med.uni-goettingen.de (G.A.M.); 5 Center for Biostructural Imaging of Neurodegeneration (BIN), University Medical Center Göttingen, D-37075 Göttingen, Germany

**Keywords:** secretome, kidney fibrosis, PPIA, transforming growth factor beta 1 (TGFβ1)

## Abstract

The secretome is an important mediator in the permanent process of reciprocity between cells and their environment. Components of secretome are involved in a large number of physiological mechanisms including differentiation, migration, and extracellular matrix modulation. Alteration in secretome composition may therefore trigger cell transformation, inflammation, and diseases. In the kidney, aberrant protein secretion plays a central role in cell activation and transition and in promoting renal fibrosis onset and progression. Using comparative proteomic analyses, we investigated in the present study the impact of cell transition on renal fibroblast cells secretome. Human renal cell lines were stimulated with profibrotic hormones and cytokines, and alterations in secretome were investigated using proteomic approaches. We identified protein signatures specific for the fibrotic phenotype and investigated the impact of modeling secretome proteins on extra cellular matrix accumulation. The secretion of peptidyl-prolyl cis-trans isomerase A (PPIA) was demonstrated to be associated with fibrosis phenotype. We showed that the in-vitro inhibition of PPIA with ciclosporin A (CsA) resulted in downregulation of PPIA and fibronectin (FN1) expression and significantly reduced their secretion. Knockdown studies of PPIA in a three-dimensional (3D) cell culture model significantly impaired the secretion and accumulation of the extracellular matrix (ECM), suggesting a positive therapeutic effect on renal fibrosis progression.

## 1. Introduction

Cellular proteins exported into extracellular fluids represent a major class of proteins, collectively referred to as the secretome [1,2]. The cell secretome consists of proteins that are secreted or shed from the cell surface, as well as intracellular proteins released into the extracellular environment due to vesiculation, cell lysis, apoptosis, and/or necrosis. The secretome can accurately reflect the functional state of secreting cells at specific time points [3] and is associated with broad cellular processes, including homeostasis, developmental regulation, proteolysis, immune defense, development of the extracellular matrix (ECM), signal transduction, and cell adhesion [4,5,6,7,8,9].

The mechanisms initiating kidney fibrosis are essential for wound healing. In the pathological case, these normal wound healing processes get out of control triggering inflammation, migration, myofibroblasts activation, extensive deposition of extra cellular matrix, and tissue injury [10,11,12,13]. Fibrosis is thereby an aberration of the tissue regeneration and inflammation, and represents a common pathway for the majority of chronic and progressive kidney diseases [10]. Renal fibrosis and other fibrotic diseases share many pathophysiological principles but the major driving process is the cross-talk between the different cell types, which trigger and sustain fibrosis [14,15,16,17]. The transition of kidney cells during fibrosis does not only result in extensive production and secretion of ECM but also in global alteration in the composition of secreted proteins [18], which is reflected in the secretion of a broad range of proinflammatory and profibrotic paracrine mediators, such as transforming growth factor beta (TGFβ) [18]. Such secreted mediators and amplifiers of fibrosis are potential therapeutic targets in the management of organ fibrosis. Due to the central role of secreted proteins in organ disease onset and progression, secretome investigations have received considerable in recent years. In the present study, we examined the impact of profibrotic stimulation of renal fibroblasts and their transition on cell secretome composition. Furthermore, we investigated the impact of the regulation of fibrotic signature proteins on the accumulation of the ECM and fibrosis progression.

## 2. Materials and Methods

### 2.1. Cell line and Culture Procedure

Human renal fibroblast cell lines TK173 and TK188 were derived from a healthy human kidney and a kidney with interstitial fibrosis, respectively [19]. The immortalization of the cell lines was achieved by transfecting them with the plasmid pSV3gpt from SV40. Both cell lines were maintained as monolayer culture as described earlier [20,21]. Briefly, the TK173 and TK188 cell lines were maintained as a monolayer culture in 75 cm^2^ tissue culture flasks (Falcon) in Dulbecco’s modified Eagle’s medium (DMEM, Thermo Fisher Scientific, Carlsbad, CA, USA). Then, 10% fetal calf serum (FCS, Gibco), 1% l-glutamine (Sigma, Steinheim, Germany), and 1% penicillin/streptomycin (Gibco) were added as supplements to the medium. The cells were passaged at 85–90% confluency.

### 2.2. Cell Culture of Renal Fibroblasts on Type I Collagen Gels

For the preparation of a collagen I-coated surface, collagen I solution 5 mg/mL (Collagen I, rat tail, Gibco) was diluted with 1× phosphate-buffered saline (PBS) to achieve a final concentration of 2.5 mg/mL. The prepared solution was added to cell culture dishes and incubated for 1 h at room temperature. After polymerization, the remaining solution was removed, and the dishes were rinsed three times with 1× PBS prior to loading cells.

### 2.3. FCS-Free Cell Culture and Cytokine/Hormone Treatment Experiments

To investigate the secretome alteration under profibrotic agents’ treatment, TK173 and TK188 cells were grown to sub-confluency (~70%) in 75 cm^2^ culture flasks. The culture medium was removed, and the cells were washed with phosphate-buffered saline (PBS, Gibco) and incubated for a further 24 h in 10 mL FCS free culture medium. During the first 10 h of the incubation period, the medium was regularly changed every 2 h to eliminate the traces of FCS, which might contaminate the secretome. For the cytokine treatment, purified human TGFβ1 (5 ng/mL), Platelet-derived growth factor Platelet-derived growth factor( PDGF) (7 ng/mL) (R&D Systems, Minneapolis, MN, USA), or the hormone Angiotensin II (ANG II) (5 μM) (Sigma-Aldrich, St Louis, MO, USA), were respectively added to the medium, and the cells were incubated at 37 °C in a humidified atmosphere with 5% CO_2_. Conditioned media containing the secretome were harvested after 72 h of incubation. 

### 2.4. Protein Extraction and Precipitation

To enrich the secretome the sample volume was reduced by carrying out an ultrafiltration step at 4000× *g* (Vivacell 100 centrifugal filter device, 5 kDa molecular weight cut-off; Sartorius, Göttingen, Germany). The resulting samples (500 µL volume) were subjected to a chloroform-methanol precipitation according to Wessel and Flügge [22]. The obtained protein pellet was dissolved in urea buffer (8 M urea, 1% (*w*/*v*) 3-[(3-Cholamidopropyl)dimethylammonio]-1-propanesulfonate hydrate, (CHAPS), 0.2% ampholytes pH 5–8 for 11 cm immobilized pH gradient (IPG) strips, 15 mM Dithiothreitol (DTT). Samples were used immediately or stored at −80 °C until use.

The protein estimation was performed according to Bradford [23] using the Bio-Rad protein assay (Bio-Rad, Hercules, CA, USA).

### 2.5. Two-Dimensional Gel Electrophoresis (2-DE)

The separation and illustration of the secretomes was carried out using 2-DE. The 2-DE was performed according to our established protocol [20]. The secretomes from cells treated with either ANG II, PDGF, or TGFβ1 and from control cells were prepared as described above and 150 µg proteins from each treatment and control were diluted in rehydration buffer (8 M urea, 1% (*w*/*v*) CHAPS, 0.2% ampholytes pH 5–8 for 11 cm IPG strips, 15 mM DTT, and a trace of bromophenol blue). The proteins were separated in the first dimension according to their isoelectric point on immobilized pH gradient (IPG) strips (pH 5–8, Bio-Rad). After the first-dimension separation and a subsequent equilibration step, the proteins were separated in a second dimension according to their molecular weight on a 12% SDS-PAGE. The 2-D gels were subjected to a fixation step (50% methanol and 12% acetic acid overnight) prior to staining with Flamingo fluorescent protein gel stain (Bio-Rad) for 5 h. The stained gels were then scanned at 50 µm resolution on a Fuji- FLA5100 (Fuji Photo, Kanagawa, Japan).

### 2.6. Image Analysis

Fluorescent images were acquired in 16-bit TIFF files format. The 2-DE analysis software Delta2D (Decodon, Greifswald, Germany) was used to match the protein spots across the gels and for normalization. The quantification of the protein level was achieved based on the average spot volume ratio and the significance of protein regulation was confirmed by Student’s *t*-test, and statistical significance was assumed for *p*-values less than 0.05. For processing the regulated proteins for mass spectrometric analysis, the 2-DE gels were then post-stained with colloidal Coomassie blue (Roti-Blue, Carl Roth) overnight.

### 2.7. In-Gel Digestion and Mass Spectrometry Analysis of Protein Spots

Manually excised plugs were subjected to automated in-gel digestion and peptide extraction followed by mass spectrometric protein identification as described previously [20,24]. The resulting peptide samples were dissolved in 0.1% formic acid and processed for mass spectrometric analysis as described earlier [20]. Alternatively, the peptide digest was analyzed on a Rapiflex-MALDI-TOF/TOF mass spectrometer (Bruker, Bremen, Germany). Data were searched against mass spectrometry protein sequencing database (MSDB) and Swiss-Prot databases through Mascot search engine using a peptide mass tolerance of 20 ppm (parts per million) and fragment tolerance of 50 mmu (millimass unit). Protein identifications with at least two peptides sequenced were considered significant.

### 2.8. Bioinformatics

The classification of the identified proteins according to their main known/postulated functions was carried out using database for annotation, visualization and integrated discovery (DAVID) bioinformatics [25]. The official gene symbol was used to investigate and categorize the Gene Ontology (GO) annotations (biological processes and molecular functions). Protein lists from each cell line under the different conditions were analyzed with the Software Tool for Rapid Annotation of Proteins (STRAP, http://www.bumc.bu.edu/cardiovascularproteomics/strap/) to classify them by GO annotations. 

### 2.9. Western Blot Analysis

Western blot analysis was carried out to confirm the proteomics data and to investigate the effect of the cytokine/hormone treatments on the level of the protein of interest. For our Western blot analysis the protocol of Towbin et al. [26,27] was adapted. First, 50 µg of the cell extracts or secretome proteins were subjected to a denaturation step with Laemmli buffer and loaded on a 12% SDS-gel. Rabbit anti-fibronectin (FN1) (Sigma), rabbit anti-alpha smooth muscle actin (Abcam), mouse anti-peptidyl-prolyl cis-trans isomerase A (PPIA) (Abcam), and mouse anti-β-actin (Sigma), as the primary antibodies were diluted in blocking buffer, then added to the membrane and allowed to incubate overnight at 4 °C. Molecular Probes^®^ Alexa Fluor 647 goat anti-mouse IgG antibody or Alexa Fluor 647 goat anti-rabbit IgG were used as secondary antibodies. Before imaging, the blots were dried in the dark. The blot membranes were scanned at 50 µm resolution on a Fuji FLA-5100 scanner (Fuji Photo, Kanagawa, Japan) with single laser emitting excitation light.

### 2.10. 3-(4,5-dimethylthiazol-2-yl)-2,5-diphenyltetrazolium bromide (MTT) Cell Viability Assay

To quantify the cell viability of the kidney fibroblasts under different treatment conditions, the non-radioactive colorimetric assay from Roche was used (Cell Proliferation Kit I, Roche Applied Science, Mannheim, Germany) according to the manufacturer’s instructions. TK173- or TK188-cells were plated in 100 µL of medium at a concentration of 6 × 10^3^ cells per flat-bottomed well in 96-well plates (tissue culture grade, Falcon). After overnight incubation, the medium was replaced with standard media containing the appropriate treatment: in the first experiment increasing concentration of ciclosporin A and FK506 (0.5 µM, 1 µM, 5 µM, 10 µM, 20 µM, 30 µM, 60 µM, and 80 µM) or siRNA against PPIA were used and the treatment was performed for 72 h; in the second experiments the cells were treated with TGFβ1 alone (5 ng/mL) or with TGFβ1 in combination with either ciclosporin A, FK506 (100 nM and 1 µM) or siRNA against PPIA for 24, 48, and 72 h. Thereafter, the cell viability was quantified using a plate reader by light absorbance at a wavelength between 550 and 600 nm with a reference wavelength >650 nm.

### 2.11. Analysis of Apoptosis by Flow Cytometry

To identify and quantify apoptotic cells, the samples were stained with recombinant Alexa Fluor 488-conjugated Annexin V and propidium iodide (PI) (PubChem CID: 104981) using cell death detection kit (Thermo Fisher Scientific, Carlsbad, CA, USA) according to the manufacturer’s instructions. In brief, after treatment with TGFβ1 alone (5 ng/mL) or with TGFβ1 in combination with either ciclosporin A or FK506 (100 nM and 1 µM) for 72 h, floating and attached cells were pooled and washed twice with PBS. Cells were incubated with 5 μL of Annexin V- Fluorescein isothiocyanate (FITC) and 2 μL of PI in the dark for 15 min at room temperature. Cells were analyzed on a fluorescence-activated cell sorting (FACS) Calibur (BD Biosciences, San Jose, CA, USA). The fraction of cells in each quadrant was calculated using Cell-Quest software (BD Biosciences).

### 2.12. Indirect Immunofluorescence Staining

To monitor the effect of the treatments on the cell phenotype, TK173 and TK188 cells were cultivated overnight in 8-well chamber slides coated with high-quality sterile polylysine (30,000 cell/well). The FCS-free cell culture and the cell treatment were performed as described above. After the treatments, the medium was removed, and the cells were washed twice with PBS buffer. Fixation of the cells was carried out for 20 min at 25 °C with 4% paraformaldehyde in PBS. Non-specific binding sites were blocked with 10% goat serum in 1% BSA (BSA/PBS) (Sigma-Aldrich, St. Louis, MO, USA) for 60 min at 37 °C. The incubation with the primary antibodies was carried out overnight at 4 °C. Alexa Fluor 488-conjugated goat anti-mouse-antibody or Alexa Fluor 555-conjugated goat anti-rabbit-antibody were used as secondary antibodies. The incubation with the secondary antibody was performed for 60 min at 37 °C in the dark. The unbounded secondary antibody was removed with three successive wash steps with PBS-buffer for 10 min each. Thereafter, the samples were counterstained with 4,6-diamidino-2-phenylindole (DAPI) in PBS buffer for 5 min. The monitoring of the staining was performed on an immunofluorescence Laser Scanning Cytometer IX71 (Olympus, Hamburg, Germany) using the CellD 3.4 software (Olympus, Hamburg, Germany).

### 2.13. Immunofluorescence Staining of the Kidney Sections

The unilateral ureteral obstruction (UUO) mouse model for kidney fibrosis was used to investigate the impact of the fibrosis progression on the expression and distribution of the protein of interest. First, 8–12-week-old mice were anesthetized with isoflurane inhalation (2–3%), analgesia was performed by subcutaneous injection of 0.1 mg/kg body weight buprenorphine per day. The ureter was separated from the surrounding tissues and two ligatures were placed about 5 mm apart in the upper two-thirds of the left ureter to obtain reliable obstruction. The mice were sacrificed 10 days after ureteral obstruction for further analyses. All animal studies were carried out with the approval of the “Landesamt für Verbraucherschutz und Lebensmittel- sicherheit” (LAVES, Oldenburg, Germany) and the University Medical Center Göttingen, Germany. To harvest the kidneys a longitudinal incision was performed to open the abdominal cavity and to collect the kidneys. The freshly harvested kidneys were processed for fixation in paraformaldehyde and embedding.

The immunostaining of deparaffinized and rehydrated sections was performed. Following an antigen retrieval pretreatment in 0.01 M citric acid using a steamer for 25 min, the sections were blocked with 10% goat serum in PBS for 1 h and incubated with primary antibodies (anti-PPIA, anti-FN1, Anti-Col1) overnight at 4 °C. The primary antibodies were detected with fluorescence Alexa 555-conjugated goat anti-rabbit or Alexa 488-conjugated goat anti-mouse secondary antibodies (Invitrogen) as recommended. The slides were rinsed and mounted with Vectashield 4,6-diamidino-2-phenylindole (DAPI) (Vector Laboratories) to visualize the nuclei. Microscopic analyses of the stained tissue sections were performed on an immunofluorescence Laser Scanning Cytometer IX71 (Olympus, Hamburg, Germany) using the CellD 3.4 software (Olympus, Hamburg, Germany).

### 2.14. siRNA Mediated Knockdown of PPIA

For investigating the effect of expression regulation of PPIA on its secretion and ECM accumulation, siRNA-mediated gene knockdown was carried out. For this purpose, 4 × 10^6^ cells were transfected with siRNA oligonucleotide specific for the knockdown of human PPIA expression. The silencer select oligos from Life Technologies (Carlsbad, CA, USA) were purchased (sense: 5′AGGUCCCAAAGACAGCAGAtt3′, antisense: 3′UCUGCUGUCUUUGGGACCUtg-5′); as the control for knock-down efficiency, non-specific controls from Eurofins MWG Operon (Munich, Germany) were used. Fibroblast cells with 70% confluence were transfected with the PPIA siRNA using transfection reagent, Lipofectamine 2000 (Invitrogen) according to the manufacturer’s protocol. The transfection reagent was removed after 8 h and replaced with FCS free culture medium. For the cell treatment the medium was supplemented with TGFβ1.

### 2.15. Protein Co-Immunoprecipitation

For protein–protein interaction studies, immunoprecipitation (IP) using a protein G-Agarose matrix was performed. Cell secretome was harvested from TK173-treated/control cells as described above and concentrated to 500 µL. Then, 100 µg secretome proteins were used for immunoprecipitation. The samples were adjusted to 1 mL with Tris-buffered saline (TBS) buffer and 5 µL monoclonal anti-PPIA or IgG were added. The samples were incubated overnight at 4 °C under agitation, and 50 µL protein G-Agarose matrix (Roche) was added to each sample. The mixture was incubated at 4 °C under rotary agitation for 3 h. The samples were centrifuged, and the supernatant removed. The Agarose matrix was washed three times with Tris-buffered saline with Tween20 (TBST) buffer. The supernatant was removed and 30 μL of 2× loading buffer were added. The samples were boiled at 95 °C for 5 min for protein denaturation and separation from the protein G-Agarose. The resulting samples were centrifuged, and the supernatant was used to run an SDS-PAGE.

### 2.16. In-Gel Digestion, Mass Spectrometric Analysis, and Identification of the Potential Interaction Partners of PPIA

The eluted proteins were prepared for tryptic digestion by a short separation on an SDS-PAGE. Proteins were visualized by colloidal Coomassie staining and the gel lanes were cut into three equally-sized gel pieces. In-gel digestion of proteins, separation of tryptic peptides by liquid chromatography (LC), and label-free quantification of proteins by mass spectrometry (MS) were essentially performed as described [28,29].

Briefly, tryptic peptides were dried down in a vacuum centrifuge, re-dissolved in 0.1% trifluoro acetic acid, and spiked with 2.5 fmol/μL of Hi3 Escherichia *coli* standard (Waters Corporation, Milford, MA, USA) for quantification purposes. Nanoscale reversed-phase LC separation of peptides was performed with a nano-Acquity ultra performance liquid chromatography (UPLC) system equipped with a Symmetry C18, 5 μm, 180 μm × 20 mm trap column and an ethylene bridged hybrid (BEH) C18, 1.7 μm, 75 μm × 100 mm analytical column (Waters Corporation). Peptides were separated over 60 min at a flow rate of 300 nL/min with a linear gradient of 1–45% mobile phase B (acetonitrile containing 0.1% formic acid) while mobile phase A was water containing 0.1% formic acid. Mass spectrometric analysis of tryptic peptides was performed using a Synapt G2-S quadrupole time-of-flight mass spectrometer in the ion mobility-enhanced data-independent acquisition (DIA) mode with drift time-specific collision energies. Continuum LC-MS data were processed using Waters ProteinLynx Global Server (PLGS) version 3.0.3 and database searches were performed against the UniProtKB/Swiss-Prot human proteome (release 2019-02, 20,415 entries) to which the sequence information for *E. coli.* Chaperone protein ClpB, porcine trypsin, and the reversed sequence of each entry was added. The false discovery rate (FDR) for protein identification was set to 1% threshold.

For post-identification analysis, the freely available software ISOQuant (http://www.isoquant.net) was used to merge the three LC-MS datasets per gel lane and to calculate the absolute in-sample amounts for each detected protein according to the TOP3 quantification approach. Protein amounts were normalized to the amount of bait protein detected and were considered as candidate interactors when either uniquely appearing in the treated sample or when showing an enrichment factor of at least 2-fold over the amount in the control sample.

### 2.17. Data Analysis

Analyses and quantification of the 2-DE images were performed using Delta2D 3.4 (Decodon, Braunschweig, Germany). The quantification of protein level is based on the average of the spot volume ratio. Spots whose relative expression is changed at least 2-fold (increase or decrease) between the compared samples were considered to be significant. Student’s *t*-test was carried out, and statistical significance was assumed for *p*-values: * *p* < 0.05, ** *p* < 0.01, *** *p* < 0.001.

To quantify the Western blots and to compare the protein levels between the samples, ImageJ software (https://imagej.nih.gov/ij/) was used. GraphPad prism version 5 was used for graphical presentation and analysis by either Student’s t-distribution or one-way ANOVA. The results are presented as the mean ± SD of at least three or more independent experiments. Differences were considered statistically significant when *p <* 0.05.

## 3. Results

### 3.1. Enrichment of Secretome Proteins: Protocol Optimization

Contamination of cell culture supernatant with residual proteins from FCS is one of the main challenges when targeting the cell secretome from cultured cell lines. Even minor contaminations with protein-rich FCS may easily mask some proteins of interest. In addition, cell culture is unavoidably accompanied by cell death. Consequently, significant amounts of cytoplasmic proteins may be released into the secretome, thereby concealing secreted proteins. To minimize the background from cytosolic- and FCS-proteins in secretome, we optimized a protocol to adjust the experimental conditions to reduce cell mortality and FCS-derived contamination, thereby achieving high purity for further analysis. Cell mortality was kept to a minimum by reducing the serum deprivation time to 24 h before the start of each experiment. The serum contamination was reduced by washing the cells five times at 2 h intervals before final incubation with the appropriate treatment. The supernatants were collected after every medium change and the proteins were isolated and processed either for 1D-PAGE or for 2D-PAGE and subsequent protein identification using mass spectrometry. As documented in the Supplemental Data (Figure S1A–F) several washing steps were required to remove the remaining FCS-proteins. The optimized protocol resulted in an enrichment of secreted proteins and in effective reduction in contaminating FCS-proteins (Figure S1G).

### 3.2. Transformation of Renal Cell Fibroblast toward Fibrosis Phenotype Results in Significant Alteration of Cell Secretome

Secreted proteins play a key role in several physiological processes and many diseases are associated with alteration in secretome quality and quantity. In the present study, we aimed to explore and identify the secretome alteration that accompanies the renal fibroblast transformation toward fibrosis phenotype. For these reasons, human renal fibroblast TK173 was treated with different pro-fibrotic agents (TGFβ1, PDGF, or ANG II) and the alterations in their secretome were investigated together with TK188 secretome using comparative proteomic analyses. 2-DE maps were generated from each secretome in triplicate and a comparative protein spot quantification was carried out.

**Figure 1:**
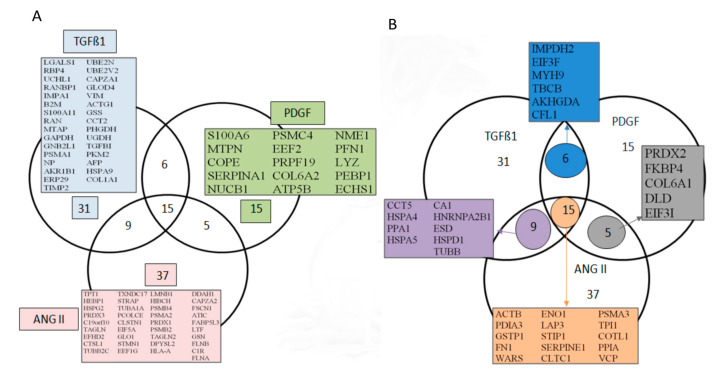
Mapping renal fibroblasts (TK173) secretome after stimulation with transforming growth factor beta 1 (TGFβ1), Angiotensin II (ANG II), and Platelet-derived growth factor (PDGF). Venn diagrams compare the gene names of the identified proteins in TK173 cells secretome. (**A**) Gene names of proteins identified under one treatment and not others. (**B**) Gene names of proteins found to be shared either between all three treatments or between two treatments.

Comparative image analysis of 2D maps from the control and TGFβ1 treated TK173 cell secretomes showed alterations of the secretion of a large number of proteins (Figure S2A,B). This reveals the significant metabolic and physiologic changes that TGFβ1 induced in the treated fibroblasts. Similar effects on secretome were also observed when the fibroblasts were stimulated with ANG II or PDFG (Figure S2C,D). Differentially secreted protein spots were excised from the 2-DE gels and processed for identification. All identified proteins were numbered and indicated on the corresponding 2-DE map (Figure S2C,D). Complete information for each of the identified proteins including protein gene name, Swiss-Prot accession number, mass average, peptide mass fingerprinting (PMF) score, and MS/MS score are provided in Tables S1–S3 for TGFβ1, ANG II, and PDGF treatments, respectively. 2-DE combined with peptide sequence analysis and database searches yielded a panel of protein sets differently secreted in TK-173 cells under TGFβ1, PDGF or ANG II treatment. We respectively identified 167 [4], 104, and 108 proteins of which 61, 41, and 66 were non-redundant proteins (Figure 1A,B). Among the identified proteins, 15 were secreted under all three treatments (Figure 1B). 

### 3.3. Bioinformatics Analyses of the Secreted Proteins

To understand the potential role of the secretome proteins in cell stimulation and fibrosis, the identified proteins were subjected to bioinformatic analyses. Comprehensive lists of proteins were compiled to provide a spatial context with localization of the proteins in the sub compartments using software tool for researching annotations of proteins (STRAP) cellular component GO term annotations. The GO analysis of these datasets showed a clear enrichment for extracellular proteins. As shown in Figure S3A, a large percentage of the identified secretome proteins (for all three treatments: TGFβ1, ANG II, and PDGF) were classified as secreted, plasma membrane or cell surface proteins and could be attributed to classical and non-classical secretory pathways. The bioinformatic analysis revealed that the secretomes also contained cytoplasmic and nuclear proteins. However, it is known that a number of intracellular proteins may also have extracellular localization.

Bioinformatic classification based on the STRAP biological function analysis shows that the differentially externalized proteins are involved in several important biological processes. The major functions of these proteins are related to regulation as well as cellular and metabolic categories (Figure S3B).

### 3.4. Secretion of Peptidyl-Prolyl Cis-Trans Isomerase A as a Common Reaction in Activated Renal Fibroblasts

**Figure 2:**
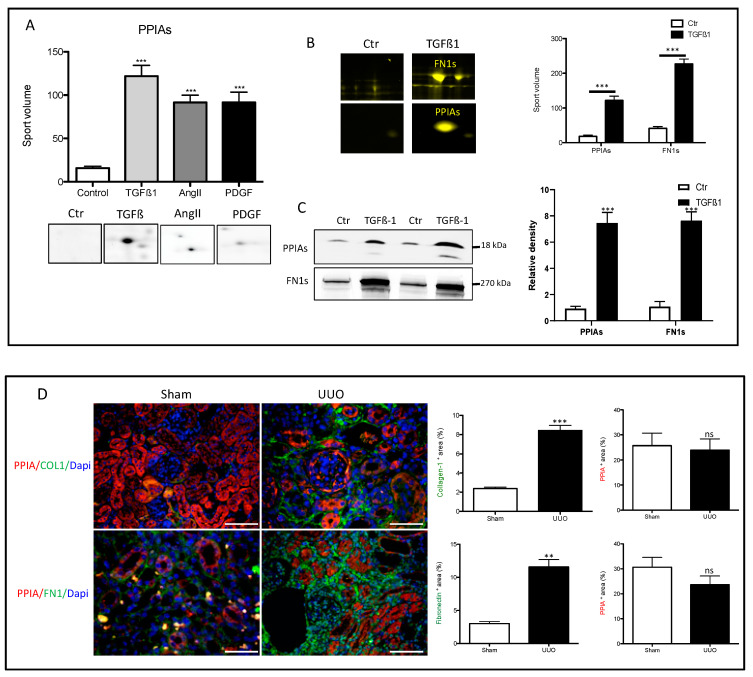
High secretion levels of peptidyl-prolyl cis-trans isomerase A (PPIA) and fibronectin (FN1) in stimulated renal fibroblasts cell lines. (**A**) Two-dimensional gel electrophoresis separation and quantification of PPIA secretion under different treatments. Spot volume quantification of PPIA under the three different treatments and control. The protein quantification in secretome is given in the form of bar diagrams. Results are given as the mean ± SD of the percentage volume of spot as quantified by two-dimensional gel electrophoresis (2-DE) software. PPIA showed significant differences in secretion level between the three different treatments and the control (*p <* 0.05). (**B**) 2-DE quantification of PPIA and FN1 secretion level in TGFβ1-treated renal fibroblasts. (**C**) Western blot analysis and quantification of the PPIA and FN1 level in secretome of the control and TGFβ1-treated renal fibroblasts cell lines. (**D**) Mice were challenged with unilateral ureteral obstruction (UUO). Kidney sections from UUO and sham were stained with antibodies against PPIA combined either with COL1 or FN1. Area positive for PPIA, or COL1 or FN1 was assessed. Data are presented as mean ± SD. ** *p* < 0.01, *** *p <* 0.001. Scale bar: 50 µm

Comparative analyses of TK173 secretome under TGFβ1, ANG II, and PDGF showed 15 proteins that were identified in the secretome of all three treatments but not in the control (Figure 1B). Among the 15 proteins peptidyl-prolyl cis-trans isomerase A (PPIA) also called cyclophilin A, or cyclosporine A-binding protein was found to be highly secreted (PPIAs) upon treatments with the pro-fibrotic agents (Figure 2A,B). Parallel to high level secretion of PPIAs, the stimulated fibroblasts secreted a high level of fibrosis markers (e.g., extracellular matrix proteins like fibronectin (FN1)) (Figure 2B) supporting cell transformation toward fibrosis phenotype. Western blot analyses confirmed the high level of PPIAs and FN1s in the secretome of TGFβ1-treated fibroblasts (Figure 2B,C). Similar results could be observed when we investigated the secretome of the fibrotic cell line TK188, where the level of PPIAs was significantly higher compared to the level in TK173 secretome (Figure S4A,B) suggesting a potential role of PPIA in renal fibrosis. PPIA is a member of the peptidyl-prolyl cis-trans isomerase family (PPIases), foldase proteins that catalyze the cis-trans isomerization of the peptide bonds. Due to its role in protein folding, the PPIA can regulate a number of biological and pathological processes [30,31]. Investigation of the PPIA expression and distribution in the kidney, showed a tubular distribution of PPIA (Figure 2D). The challenging of the mice kidney with UUO did not impact the tissue level of PPIA, and quantification of PPIA in UUO and sham kidney sections showed a non-significant decrease in the expression of PPIA in UUO kidney (Figure 2D). In contrast to sham the obstructed kidneys increased the expression of PPIA in glomeruli (Figure 2D).

### 3.5. Secretion of PPIA Increased Significantly during Fibrosis Progression

**Figure 3:**
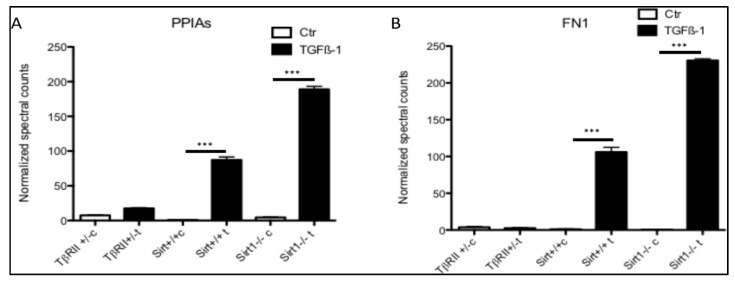
Quantitative analysis of PPIA secretion in Sirt^−/−^ and TβRII^+/−^ mice primary cell secretomes from our former published studies [4,32,33]. Primary endothelial cells were isolated from Sirt^+/+^, Sirt^−/−^, TβRII^+/+^, and TβRII^+/−^ mice kidneys (*n* = 3 for each mouse genotype) and treated with TGFß1. Supernatant from treated cells and controls were collected and the secretomes were analyzed using mass spectrometry. (**A**) The quantitative analyses showed that PPIA was highly secreted in Sirt^+/+^, Sirt^−/−^, and TβRII^+/+^ upon TGFβ1 treatment, but not in TβRII^+/−^. Moreover, the secretion level in the Sirt^−/−^ primary cells was two times higher than in treated Sirt^+/+^ cells. (**B**) Quantitative presentation of the secretion level of FN1 in Sirt^−/−^ and TβRII^+/−^ primary cells upon TGFβ1 treatment. The normalized spectral counts were used for protein level quantification (*** *p* < 0.001).

To investigate whether the PPIA secretion is tied to fibrosis progression, we analyzed the proteomic data from our former published study [4,32,33] with the focus on PPIA secretion. For this purpose, two strains of mutant mice with endothelial cells either lacking exon 4 encoding for the deacetylase activity of sirtuin 1 (Sirt1^−/−^) or deficient in TGFβ receptor II (TβRII^+/−^) were used. The Sirt1^−/−^ mice are prone to develop fibrosis, whereas TβRII^+/−^ mice are protected from fibrosis. The Sirt1^−/−^ mouse model has a broad applicability because endothelial Sirt1 deficiency is constantly accompanied by a broad range of chronic renal diseases. Sirt1-deficient endothelial cells exhibit all criteria of dysfunction especially an exaggerated fibrotic response. In contrast, the TβRII^+/−^ model has no distinctive phenotype under basal conditions but develops less fibrosis after induction of unilateral ureteral obstruction or chronic phase of folic acid nephropathy [32,33,34]. In view of the fact that Sirt1^−/−^ mice have an exaggerated, endothelium initiated fibrotic response, and that TβRII^+/−^ are protected from fibrosis, we first examined the secretion of PPIA under basal conditions and then following a fibrogenic stimulus (TGFβ1) in the cells derived from these mice. Renal microvascular endothelial cells (RMVEC) from control wild-type Sirt1^−/−^ and TβRII^+/−^ mice were expanded, exposed to TGFβ1 (5 ng/mL, 48 h incubation), and conditioned medium was collected. The secretome was enriched using protein precipitation and analyzed using unbiased, non-targeted MS/MS. The quantitative mass spectrometry analysis revealed that PPIA was highly enriched in the secretome of renal microvascular endothelial cells (RMVEC) obtained from wild-type and Sirt1^endo −/−^ mice upon stimulation with TGFβ1 (Figure 3A). In case of Sirt ^−/−^ the TGFβ1 treatment resulted in two times higher secretion level of PPIA than in the treated wild-type. In cells from TβRII^+/−^ no secretion of PPIA was identified. Moreover, our data also showed that the high-level secretion of PPIA is accompanied with high-level secretion of FN1 (Figure 3B). This suggests that the secretion of PPIA is a pro-fibrotic response of the cells, which is tied to fibrosis progression.

### 3.6. Effect of Ciclosporin A (CsA) and FK506 on Fibroblasts Cell Viability and Survival

In the PPIase family, besides PPIA, the FK506-binding protein (FKBP) is also known to act as receptors for immunosuppressive drugs. PPIA and FK506 binding protein (FKBP) bind to cyclosporin A (CsA) and FK506, respectively [35]. To investigate whether the PPIA upregulation and secretion represent an important aspect in fibrosis progression, PPIA inhibitory studies were carried out using CsA and FK506 combined with cytokine treatments.

**Figure 4:**
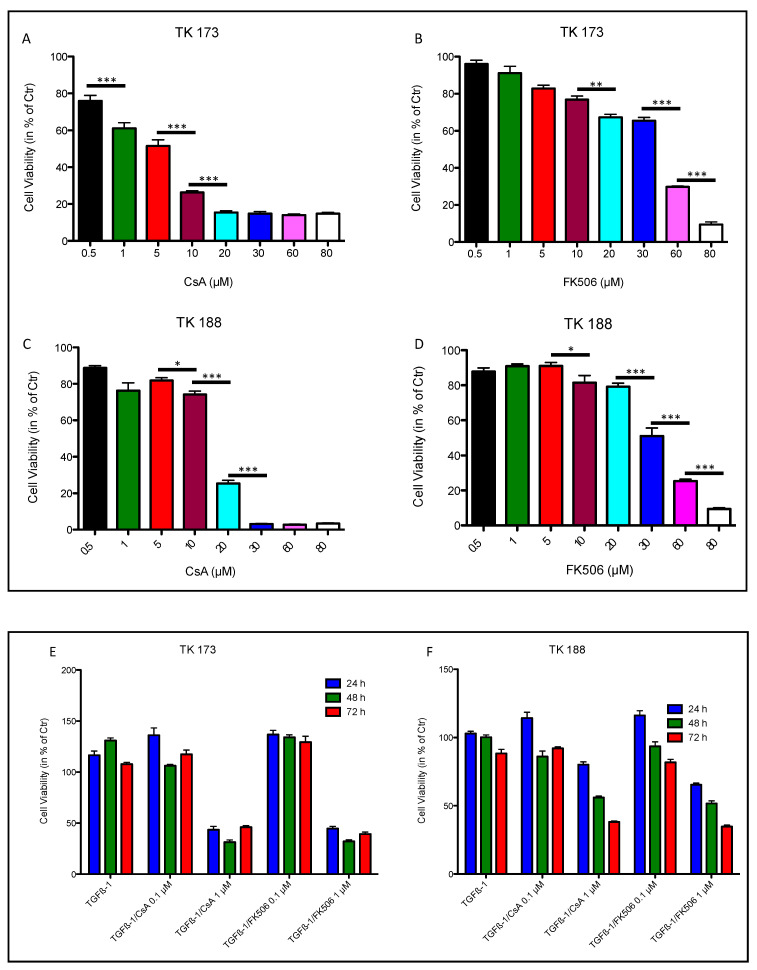
Effect of ciclosporin (CsA) and FK506 on renal fibroblasts cell line proliferation and viability. Renal fibroblast cell lines TK173 and TK188 were treated with variable (**A**) CsA or (**B**) FK506 concentrations (0.5–80 µM) and the cell viability was assessed using 3-(4,5- dimethylthiazol-2-yl)-2,5-diphenyltetrazolium bromide (MTT) assay. Concentrations >0.5 µM CsA and >10 µM FK506 significantly affect the cell viability. The fibrotic cell line TK188 was more resistant to the treatments (**C**,**D**); the viability was significantly impaired with CsA >1 µM, and FK506 >10 µM. The viability assay showed that the TK173 and TK188 survival under the TGFβ1/CsA or TGFβ1/FK506 combined treatment was dependent on the inhibitor concentration (**E**,**F**). * *p* < 0.05, ** *p* < 0.01, *** *p <* 0.001.

To assess the toxic effect of PPIase inhibitors CsA and FK506 on TK173 and TK188 cells, we monitored the response of the cells stimulated with different concentrations of CsA and FK506 over 48 h using the MTT cell viability assay. The cell viability was calculated in percent of the control (cells without treatment cultured in FCS-free medium for 48 h). As shown in Figure 4A,B, the TK173 cells react differently to CsA and FK506. In the case of CsA treatment, the TK173 cell survival correlated strongly with the concentration of the CsA; concentrations >0.5 µM resulted in a significant decrease in cell viability compared to the control, whereas lower concentrations (<0.5 µM) did not result in significant alteration in cell viability. In contrast, FK506 treatment did not significantly impact the cell survival at concentrations ≤10 µM. CsA treatment of TK188 cells resulted in a significant decrease in cell viability compared to the control and showed a concentration-dependent alteration of the cell viability (Figure 4C). Similar to TK173, the TK188 cells showed no significant changes in cell viability under treatment with low concentrations of FK506 (≤10 µM) compared to the control (Figure 4D), whereas high concentrations significantly altered the cell survival.

To address the question of whether the effect of the PPIase inhibitors CsA and FK506 on cells resulted in cell death, renal fibroblasts TK173 were treated with increasing doses of the immunosuppressive agents (5–30 µM for CsA and 15–80 µM for FK506) for 72 h. Apoptosis was then detected by Annexin V/PI staining and FACS analysis. Treatment of the cells with high concentrations of both agents resulted in an increase in apoptosis level (Figure S5A,B). The data revealed that lower concentrations of CsA may have no significant effect on fibroblasts, but when the concentration increased >15 µM the percentage of death cells also increased (Figure S5A). In case of FK506 the apoptosis level was in general lower than CsA. In contrast to CsA the apoptosis assay showed that the percentage of death cells in FK506-treated cells increased with the concentration of the treatment agent (Figure S5B).

To further study the effect of PPIA inhibition on cell transformation and fibrosis phenotype, a combined treatment with the pro-fibrotic cytokine TGFβ1 and the PPIase inhibitors CsA and FK506 was carried out. For this purpose, two different concentrations of the inhibitors (0.1 µM and 1 µM) and three different incubation times (24, 48, and 72 h) were chosen. The MTT assay showed that the cell survival under the combined treatment was more dependent on the inhibitor concentration than on incubation time (Figure 4E,F). Neither CsA nor FK506 significantly impacted the cell viability at lower concentrations (0.1 µM), whereas an increase in inhibitor concentration (1 µM) resulted in significant alteration in cell survival. The apoptosis assay confirmed the MTT data, an increase in PPIase inhibitor impacted significantly the cell survival (Figure S6A–D) in both cell lines TK173 and TK188. The apoptosis rate increased significantly, when the cells were exposed to higher inhibitor concentrations (1 µM). Consequently, based on MTT and apoptosis assay data, we chose an inhibitor concentration of 0.1 µM for further investigation to avoid the impact of apoptosis on our data.

### 3.7. Impact of PPIA Inhibition on ECM Production and Accumulation

**Figure 5:**
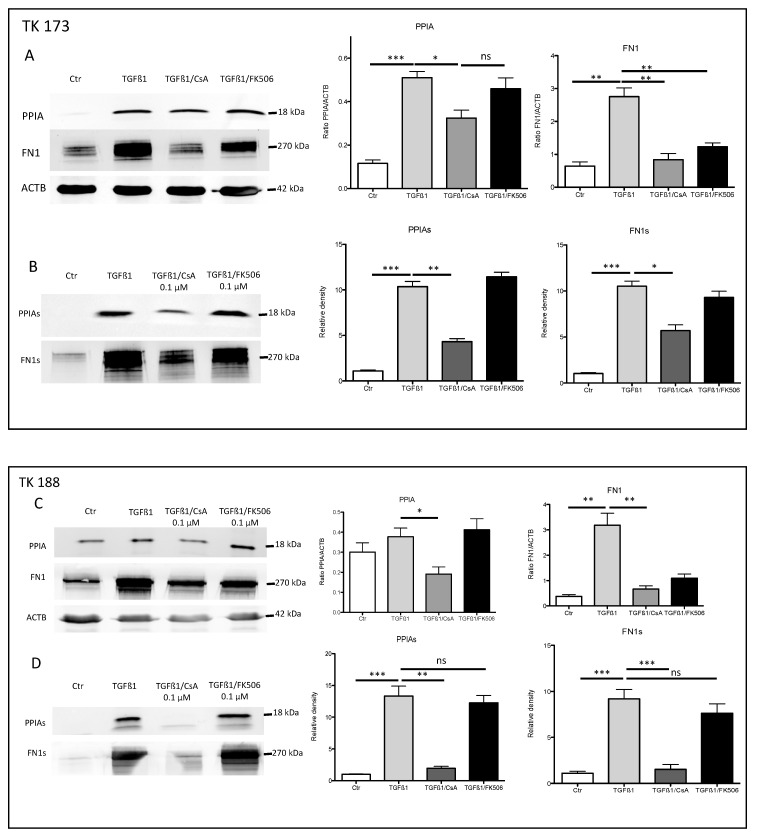
PPIA inhibition resulted in alteration of extracellular matrix (ECM) synthesis and secretion. TK173 was treated with TGFβ1 or TGFβ1 combined either with CsA or FK506 for 48 h. (**A**,**B**) The proteins were isolated from cultured cells or supernatant as described in Section 2. The Western blots were performed with antibodies against PPIA, and FN1 for secretome samples in case of cell extract beta-actin (ACTB) was used as the loading control. A bar diagram representing the quantification of the Western blot results for cell extract data is presented as the ratio of the PPIA/ACTB or FN1/ACTB (*n* = 4, * *p* < 0.05). For secretome samples, the data are presented in intensity (%). (**C**,**D**) Western blot quantification for the TK188 treatments. * *p* <0.05, ** *p* < 0.01, *** *p* <0.001.

**Figure 6:**
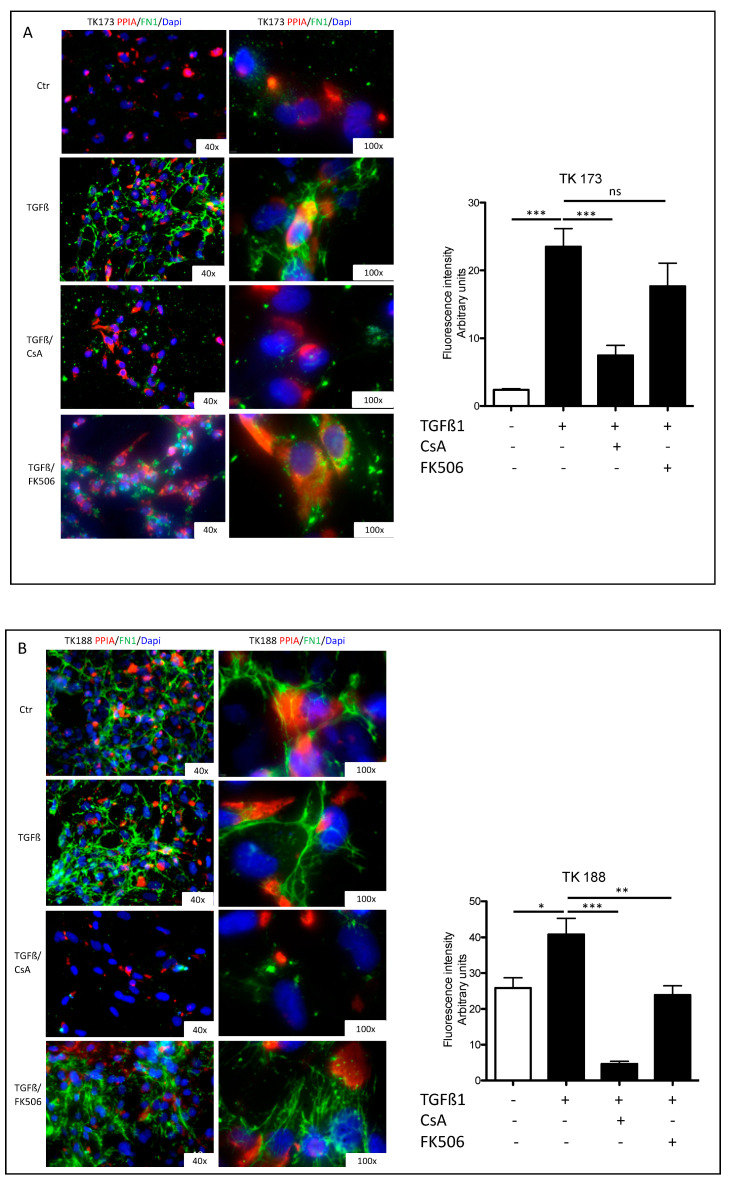
PPIA inhibition resulted in significant alteration in FN1 synthesis and accumulation. Renal fibroblasts cell lines were treated with TGFβ1 or TGFβ1 combined either with CsA or FK506 for 48 h. After the fixation with paraformaldehyde, the cells were stained with antibodies against PPIA and FN1. Fluorescence quantification (arbitrary units) is presented as a grouped bar chart with error bars. Significant differences: * *p* < 0.05, ** *p* < 0.01, *** *p* < 0.001. (**A**) TK173 left panel 400× magnification and right panel 1000× magnification; (**B**) TK188 left panel 400× magnification and right panel 1000× magnification.

Stimulation of renal fibroblasts (TK173) with TGFβ1 resulted in a significant increase of PPIA expression and secretion (Figure 5A,B). Simultaneously, the expression and secretion of ECM protein FN1 increased significantly (Figure 5A,B). Interestingly, the combined TGFβ1/CsA treatment lead on to a significant decrease in PPIA expression and secretion, which was accompanied with a significant decrease in FN1 expression and secretion suggesting a direct effect of CsA on ECM accumulation. In contrast, the combined treatment of TGFβ1/FK506 did not significantly affect the expression and secretion of PPIA (Figure 5A,B). In the case of FN1, the expression was significantly decreased in combined treatment, whereas no significant alteration in the secretion level of the protein was observed within the incubation time of 48 h (Figure 5A,B). Similar results were observed in the fibrotic kidney cell line TK188; TGFβ1 treatment increased the expression of FN1 and increased secretion of PPIA and FN1, whereas the combined TGFβ1/CsA cell stimulation decreased the expression and secretion of both proteins (Figure 5C,D). TGFβ1/FK506 combined did not significantly affect the expression and secretion of the investigated proteins in TK188 (Figure 5C,D). Immunofluorescence staining of PPIA and FN1 in kidney fibroblasts cell lines (TK173 and TK188) under the different treatments confirmed the Western blot data (Figure 6A,B). There was significant downregulation of PPIA and FN1 under CsA treatment despite the pro-fibrotic effect of TGFβ1. 

### 3.8. Co-Immunoprecipitation Revealed Interaction of PPIA with ECM and Inflammatory Proteins

PPIases exhibit interaction with ECM proteins favorizing their folding and accumulation [36,37]. Our data suggest a potential role of PPIA in ECM secretion and accumulation. To support this suggestion a mass spectrometry and label free based identification and quantification of interaction partner of PPIA were performed. Co-immunoprecipitation was carried out from secretome of TGFβ1-treated TK173 cells and their non-treated controls, using anti-PPIA antibody to isolate PPIA with potential interaction partners. The PPIA and the binding proteins were separated by 1D SDS-PAGE and identified and quantified using mass spectrometry and data search as described in Section 2. Table 1 shows the identified potential interaction partner of PPIA and their level of enrichment in the secretome of TGFβ1-treated fibroblasts. We listed proteins, which were identified uniquely or enriched at least two-fold in TGFβ1 secretome compared to the control. In addition to several known interaction partners of PPIA like CD147 (Basigin, EMMPRIN), other proteins were identified for the first time as potential interaction partners for PPIA (Table 1, Table S4). Interestingly, ECM proteins, especially fibronectin (FN1), collagen alpha-1(VI) (COL6A1), and collagen alpha-2(VI) (COL6A2) were identified as interaction partners of PPIA only in the secretome of TGFβ1-treated cells, corroborating the role of PPIA in the folding and accumulation of ECM proteins. Another group of proteins, which were enriched in the secretome of TGFβ1 stimulated cells were inflammatory proteins. SERPINE1, ICAM1, ITGA5, ITGAV, ALCAM, THBS1, and EGFR are important players in the inflammation process and were found to be enriched in the secretome of activated fibroblasts (Table 1).

### 3.9. siRNA Mediated Downregulation of PPIA and Its Impact on Cell Transformation and EMC Expression and Accumulation

**Figure 7:**
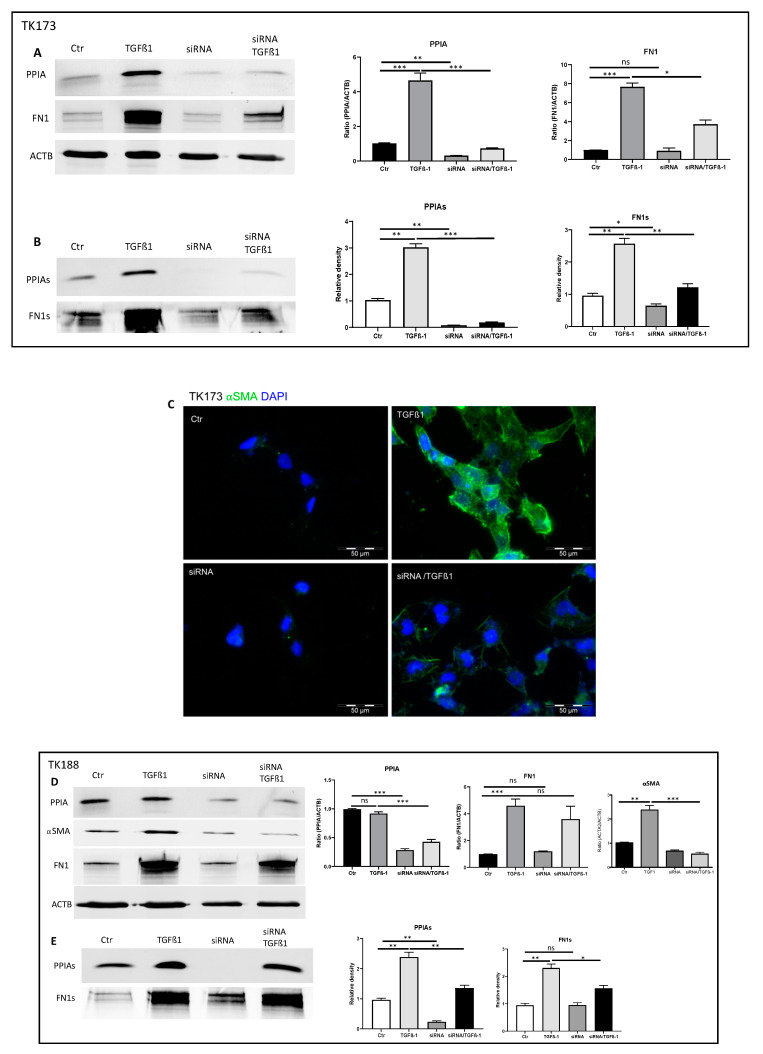

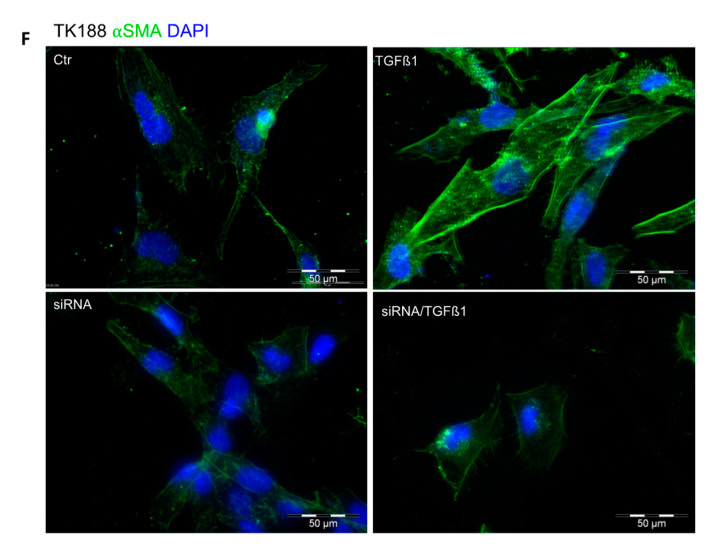
PPIA downregulation resulted in significant alteration of PPIA secretion, ⍺SMA expression, and ECM synthesis and secretion. To achieve 3D cell culture conditions, renal fibroblasts were cultured on collagen I-coated surface, TK173 and TK188 controls and siRNA treated were stimulated with TGFβ1 for 48 h. (**A**,**B**,**D**,**E**) Proteins were enriched from TK173 and TK188 cell extracts or supernatant as described in Section 2. The Western blots were performed with antibodies against PPIA, ⍺SMA, FN1. In case of cell extract ACTB was used as a loading control. Bar diagram representing the quantification of the Western blot results for cell extract data is presented as the ratio of the PPIA/ACTB or FN1/ACTB (*n* = 4, * *p* < 0.05). For secretome samples, the data are presented in intensity (%). (**C**,**F**) Renal fibroblasts cell lines were treated with TGFβ1 or TGFβ1 combined with siRNA against PPIA for 48 h. After the fixation with paraformaldehyde, the cells were stained with antibodies against ⍺SMA. * *p* < 0.05, ** *p* < 0.01, *** *p <* 0.001 ns: non-significant.

The inhibition experiment with CsA revealed that PPIA may alter ECM synthesis and accumulation and additionally have an anti-fibrosis effect. Since CsA may bind other PPIases and the effect may not be attributed to CsA only, we combined a 3D cell culture model on collagen I with genetic approach. We used siRNA to knockdown PPIA and investigated the effect on PPIA secretion, alpha-smooth muscle actin (⍺SMA) expression, and ECM synthesis and accumulation. The siRNA treatment resulted in significant knockdown of PPIA in both cell lines (Figure 7A,D). Moreover, the knockdown of PPIA was accompanied by a significant reduction in FN1 synthesis and secretion (Figure 7A,B,D,E). The treatment of the fibroblasts with the pro-fibrotic cytokine TGFβ1 resulted in significant alteration of PPIA and ECM secretion under PPIA knock-down conditions. Moreover, we observed a significant alteration in cell transformation as evidence by investigations of ⍺SMA expression (Figure 7C,D,F). The effect was more prominent in fibroblasts from healthy kidney TK173 (Figure 7A,B,D,E). Hence our results suggest that PPIA plays an important role in cell transformation and ECM synthesis and accumulation.

## 4. Discussion

Fibrosis is a common pathological complication, which results from an aberrant response to organ injury. Fibroblast-to-myofibroblast transition, characterized by excessive ECM production and deposition, plays a critical role in fibrogenesis and count to the driving forces of renal fibrosis [38,39,40,41]. We reported that this transformation of renal cells toward fibrotic phenotype is accompanied by alteration in renal cell secretome [4,21,32,33], which may result in stimulation of other cells and amplification of fibrosis. The secretome is cell- and tissue-specific, and its composition is highly dynamic and changes in response to fluctuations in physiologic states or under pathologic conditions. The secreted proteins are involved in the control of a broad range of physiological functions [4,5,6,32], which make it an incredible source for disease biomarkers and target for therapeutic interventions. Therefore, a number of studies, including ours, emerged in recent years with the aim to identify biomarkers from secretomes and elucidate their therapeutic potential [21,32,33,34,42,43,44]. The prominent feature of the present study consists of comparative proteomic analysis of secretomes from human renal fibroblast cells stimulated with three different pro-fibrotic agents, with their untreated control and with the secretome from a fibrotic cell line. The secretomes derived from stimulated human fibroblast cells exhibited unique signatures. We detected proteins specific for the treatment type, which could potentially be responsible for or contributed to fibrosis phenotype. Other proteins were identified in two or in all three treatments. Such common secretome aberrations may represent global fibrosis aspects and are of special interest. Among the commonly identified proteins, we detected known fibrosis markers, which were highly secreted upon treatment confirming the transition of fibroblasts toward fibrotic phenotype and other proteins, which were new in the context of fibrosis. Here, we stress the focus on the role of the peptidyl-prolyl cis-trans isomerase A (PPIA), a foldase protein, in renal fibrosis. The PPIA was secreted in high amounts upon cytokine treatment especially in kidney fibroblasts treated with TGFβ1, whereas in the secretome of untreated control cells, no PPIA could be identified. PPIA belong to peptidylprolyl cis-trans isomerases (PPIases), a family of proteins which catalyze the cis to trans conversion of proline-containing peptides and are involved in folding, dynamics, and function of different proteins [45,46]. The PPIases share a common cyclophilin-like domain, a 109 amino acid long conserved peptide, whereas the rest of the polypeptide is specific for each PPIase and is responsible among others for the subcellular localization of the protein [47]. PPIA is the most abundant human PPIase and is an interaction partner of the immunosuppressive agent ciclosporin A [48]. PPIA is mainly localized in the cytoplasm but it can be secreted spontaneously or in response to oxidative stress and inflammatory stimulation [49]. In our study, the upregulation of PPIA and its secretion upon cytokines stimulation in the course of renal fibroblasts transition suggest an important role of the protein in the pathogenesis of kidney fibrosis. To investigate the importance of PPIA in pathomechanism of fibrosis, inhibition experiments were performed in renal fibroblast cells using PPIase inhibitors or a genetic approach with siRNA knockdown. The in vitro data revealed that the inhibition of PPIA impaired cell proliferation and survival. Moreover, inhibition of PPIA was accompanied by a significant reduction in ECM proteins expression and accumulation. It is known that PPIases also bind to ECM proteins, like collagen, and catalyze their folding favorizing their accumulation [36,37]. Our interaction studies identified ECM proteins especially collagen and fibronectin as interaction partners of PPIA. Moreover, inhibition of PPIA resulted in a significant decrease in accumulation of ECM proteins, thus suggesting a role of PPIA in folding and accumulation of ECM proteins and fibrosis progression. Similar effects of PPIA inhibition were observed in heart diseases, blocking extracellular PPIA significantly reduced inflammation and resulted in pronounced reduction of myocardial fibrosis [50]. The beneficial effect of PPIA inhibition was also demonstrated in an animal model of liver fibrosis [51]. It was also reported that the secreted PPIA has pro-inflammatory properties, which enable inflammatory responses via its cellular receptor CD147 also known as Basigin or EMMPRIN [52]. Moreover, different studies demonstrated the anti-inflammatory effect of PPIA inhibition [49,50,53]. Our interaction study identified CD147 (Basigin) as an interaction partner of PPIA in kidney fibroblasts. Furthermore, key player proteins in inflammation were identified as interaction partners of PPIA and were highly enriched in the secretome of TGFβ1-treated kidney fibroblasts, suggesting a potential inflammatory role of the secreted PPIA in the case of kidney fibrosis.

Our data provide evidence of the potential role of PPIA in renal fibrosis. We also demonstrated that inhibition of PPIA has a beneficial effect in kidney fibrosis phenotype.

## Figures and Tables

**Table 1:** List of proteins identified after co-immunoprecipitation with anti-PPIA antibody and mass spectrometric analysis. The identified proteins were quantified, and their amount is given in fmol. Listed are the potential interaction partners of PPIA under TGFβ1 treatment. These proteins bind to PPIA only under TGFβ1 treatment. The gene name, accession number in Swiss-Prot, molecular weight, entry, isoelectric point, and the amount in fmol are given.

Protein Name	Gene Name	Accession	Entry	IEP	MW	Ctr (fmol)	TGFβ1 (fmol)
Peptidyl-prolyl cis-trans isomerase A	PPIA	P62937	PPIA_HUMAN	7.85	18,240.64	411.95	568.08
Heterogeneous nuclear ribonucleoprotein U	HNRNPU	Q00839	HNRPU_HUMAN	5.66	91,325.94		329.21
Ubiquitin-40S ribosomal protein S27a	RPS27A	P62979	RS27A_HUMAN	10.24	18,307.10		135.32
Galectin-1	LGALS1	P09382	LEG1_HUMAN	5.15	15,057.89	61.14	103.95
E3 SUMO-protein ligase RanBP2	RANBP2	P49792	RBP2_HUMAN	5.81	362,590.78		78.65
Heat shock protein 75 kDa_ mitochondrial	TRAP1	Q12931	TRAP1_HUMAN	8.31	80,395.28	11.33	69.58
Fibronectin	FN1	P02751	FINC_HUMAN	5.36	266,217.54		64.91
Plasminogen activator inhibitor 1	SERPINE1	P05121	PAI1_HUMAN	6.79	45,117.13	5.66	56.30
Bifunctional purine biosynthesis protein PURH	ATIC	P31939	PUR9_HUMAN	6.30	65,129.21	10.66	43.70
Intercellular adhesion molecule 1	ICAM1	P05362	ICAM1_HUMAN	7.92	58,623.66	18.39	40.33
Splicing factor 3B subunit 1	SF3B1	O75533	SF3B1_HUMAN	6.69	146,572.02	2.43	36.54
Myosin-10	MYH10	P35580	MYH10_HUMAN	5.28	229,968.92	7.87	31.49
Peptidyl-prolyl cis-trans isomerase B	PPIB	P23284	PPIB_HUMAN	10.06	23,799.61	14.31	29.33
Ras-related protein Rap-1A	RAP1A	P62834	RAP1A_HUMAN	6.53	21,329.38		28.94
40S ribosomal protein S20	RPS20	P60866	RS20_HUMAN	10.71	13,486.79		27.24
Aldo-keto reductase family 1 member B1	AKR1B1	P15121	ALDR_HUMAN	6.59	36,252.64	8.73	27.09
Brain acid soluble protein 1	BASP1	P80723	BASP1_HUMAN	4.42	22,693.42	6.91	21.55
Myosin-11	MYH11	P35749	MYH11_HUMAN	5.25	228,195.03	4.50	21.10
Ras-related C3 botulinum toxin substrate 1	RAC1	P63000	RAC1_HUMAN	8.58	21,849.32		20.56
Tumor protein D54	TPD52L2	O43399	TPD54_HUMAN	5.08	22,294.78	4.45	13.04
Integrin alpha-5	ITGA5	P08648	ITA5_HUMAN	5.41	115,677.14	4.45	11.68
Cytoplasmic dynein 1 heavy chain 1	DYNC1H1	Q14204	DYHC1_HUMAN	5.99	535,145.98		10.62
Bifunctional glutamate/proline--tRNA ligase	EPRS	P07814	SYEP_HUMAN	7.00	172,187.91		9.30
CD166 antigen	ALCAM	Q13740	CD166_HUMAN	5.87	65,786.70	4.70	9.04
Serpin B12	SERPINB12	Q96P63	SPB12_HUMAN	5.22	46,675.69		8.14
Small nuclear ribonucleoprotein F	SNRPF	P62306	RUXF_HUMAN	4.43	9782.26		8.03
Integrin alpha-V OS=Homo sapiens	ITGAV	P06756	ITAV_HUMAN	5.32	117,121.56		7.89
Thrombospondin-1	THBS1	P07996	TSP1_HUMAN	4.53	133,374.73		7.83
Proteasome subunit beta type-1	PSMB1	P20618	PSB1_HUMAN	8.22	26,717.54		7.69
Protein S100-A10	S100A10	P60903	S10AA_HUMAN	7.27	11,317.21		7.46
S-phase kinase-associated protein 1	SKP1	P63208	SKP1_HUMAN	4.20	18,829.12		7.42
Splicing factor_ proline- and glutamine-rich	SEPQ	P23246	SFPQ_HUMAN	9.95	76,263.60		7.39
LIM and SH3 domain protein 1	LASP1	Q14847	LASP1_HUMAN	6.70	30,116.41		7.30
Coatomer subunit beta	COPB1	P53618	COPB_HUMAN	5.66	108,282.98		6.84
Isoleucine--tRNA ligase_ cytoplasmic	IARS	P41252	SYIC_HUMAN	5.77	145,809.96		6.35
Heterogeneous nuclear ribonucleoprotein D-like	HNRNPDL	O14979	HNRDL_HUMAN	9.96	46,608.67		6.26
Tropomyosin beta chain	TPM2	P07951	TPM2_HUMAN	4.46	32,964.85		5.84
GTPase NRas	NRAS	P01111	RASN_HUMAN	4.82	21,514.35		5.82
Cystatin-A	CSTA	P01040	CYTA_HUMAN	5.22	11,006.51		5.36
Protein Niban	FAM129A	Q9BZQ8	NIBAN_HUMAN	4.54	104,104.30		5.10
ELAV-like protein 1	ELAVL1	Q15717	ELAV1_HUMAN	9.57	36,263.04		5.07
Podocalyxin	PODXL	O00592	PODXL_HUMAN	5.16	59,091.56		4.99
Putative heat shock protein HSP 90-beta 2	HSP90AB2P	Q58FF8	H90B2_HUMAN	4.59	44,520.09		4.97
Ephrin type-A receptor 2	EPHA2	P29317	EPHA2_HUMAN	5.83	109,749.33		4.64
Collagen alpha-1(VI) chain	COL6A1	P12109	CO6A1_HUMAN	5.09	109,670.06		4.58
Transportin-1	TNPO1	Q92973	TNPO1_HUMAN	4.65	103,837.95		4.46
Phosphatidylinositol transfer protein beta isoform	PITPNB	P48739	PIPNB_HUMAN	6.47	31,825.26		4.46
Thioredoxin-dependent peroxide reductase_ mitochondrial	PRDX3	P30048	PRDX3_HUMAN	7.70	28,034.84		4.13
Putative heat shock protein HSP 90-beta 4	HSP90AB4P	Q58FF6	H90B4_HUMAN	4.45	58,891.78		4.00
Phosphoribosylformylglycinamidine synthase	PFAS	O15067	PUR4_HUMAN	5.41	146,388.37		3.94
Epidermal growth factor receptor	EGFR	P00533	EGFR_HUMAN	6.27	137,699.25		3.85
Glucosidase 2 subunit beta	PRKCSH	P14314	GLU2B_HUMAN	4.13	60,394.97		3.83
Peripherin	PRPH	P41219	PERI_HUMAN	5.21	53,765.00		3.74
Ras GTPase-activating-like protein IQGAP3	IQGAP3	Q86VI3	IQGA3_HUMAN	7.37	185,383.40		3.64
Hippocalcin-like protein 1	HPCAL1	P37235	HPCL1_HUMAN	5.03	22,427.29		3.54
Glial fibrillary acidic protein	GFAP	P14136	GFAP_HUMAN	5.26	49,937.33		3.41
Ras-related protein Ral-B	RALB	P11234	RALB_HUMAN	6.26	23,522.64		3.38
Structural maintenance of chromosomes protein 4	SMC4	Q9NTJ3	SMC4_HUMAN	6.38	147,866.56		3.34
Aminoacyl tRNA synthase complex-interacting multifunctional protein 2	AIMP2	Q13155	AIMP2_HUMAN	8.27	35,691.02		3.31
Ras suppressor protein 1	RSU1	Q15404	RSU1_HUMAN	9.23	31,540.35		3.31
Ras-related protein Rab-23	RAB23	Q9ULC3	RAB23_HUMAN	6.24	26,887.39		3.21
Collagen alpha-2(VI) chain	COL6A2	P12110	CO6A2_HUMAN	5.81	109,777.13		2.90
Structural maintenance of chromosomes protein 2	SMC2	O95347	SMC2_HUMAN	8.73	136,169.56		2.75
FACT complex subunit SPT16	SUPT16H	Q9Y5B9	SP16H_HUMAN	5.37	120,484.25		2.71
Aminopeptidase N	ANPEP	P15144	AMPN_HUMAN	5.16	109,938.95		2.62
Glycogen phosphorylase_ brain form	PYGB	P11216	PYGB_HUMAN	6.43	97,380.45		2.54
CAD protein	CAD	P27708	PYR1_HUMAN	6.03	245,322.25		2.49
DNA replication licensing factor MCM2	MCM2	P49736	MCM2_HUMAN	5.20	102,580.63		2.45
Signal transducer and activator of transcription 2	STAT2	P52630	STAT2_HUMAN	5.20	98,657.73		2.44
Valine--tRNA ligase	VARS	P26640	SYVC_HUMAN	7.37	141,730.79		2.30
Peroxidasin homolog	PXDN	Q92626	PXDN_HUMAN	6.79	167,898.08		2.23
Microtubule-associated protein 4	MAP4	P27816	MAP4_HUMAN	5.14	121,518.45		2.15
Myoferlin	MYOF	Q9NZM1	MYOF_HUMAN	5.79	236,248.81		2.13
Kinesin-like protein KIF23	KIF23	Q02241	KIF23_HUMAN	8.59	111,085.51		2.03
Procollagen-lysine_2-oxoglutarate 5-dioxygenase 2	PLOD2	O00469	PLOD2_HUMAN	6.27	85,427.10		1.45

## Supplementary Materials

The following are available online at https://www.mdpi.com/2073-4409/9/7/1724/s1, Figure S1: Secretome enrichment: Protocol optimization, Figure S2: -DE reference maps of secretomes, Figure S3: Classification of the differentially expressed proteins, Figure S4: Two-dimensional pattern of total proteins isolated from TK173, TK173/TGFβ1 and TK 188 secretomes, Figure S5 FACS analysis, Figure S6: Low concentration of CsA and FK506 did not have significant impact on cell survival under TGFβ1 treatment. Table S1: List of non-redundant proteins found to be differently secreted in the TGFβ1 treated TK173 cells compared to untreated control, Table S2: List of non-redundant proteins found to be differently secreted in the ANGII treated TK173 cells compared to untreated control, Table S3 List of non-redundant proteins found to be differently secreted in the PDGF treated TK173 cells compared to untreated control, Table S4: List of potential interaction partner of PPIA identified and quantified using mass spectrometry.
