# Supplementary material for: The Secretome Analysis of Activated Human Renal Fibroblasts Revealed Beneficial Effect of the Modulation of the Secreted Peptidyl-Prolyl Cis-Trans Isomerase A in Kidney Fibrosis
Source: Cells. 2020 Jul 18;9(7):1724. doi: 10.3390/cells9071724 (PMC7407823; doi:10.3390/cells9071724)
Supplement: Supplementary file 1 [file cells-09-01724-s001.pdf]

Supplemental Material

Supplemental Figure 1

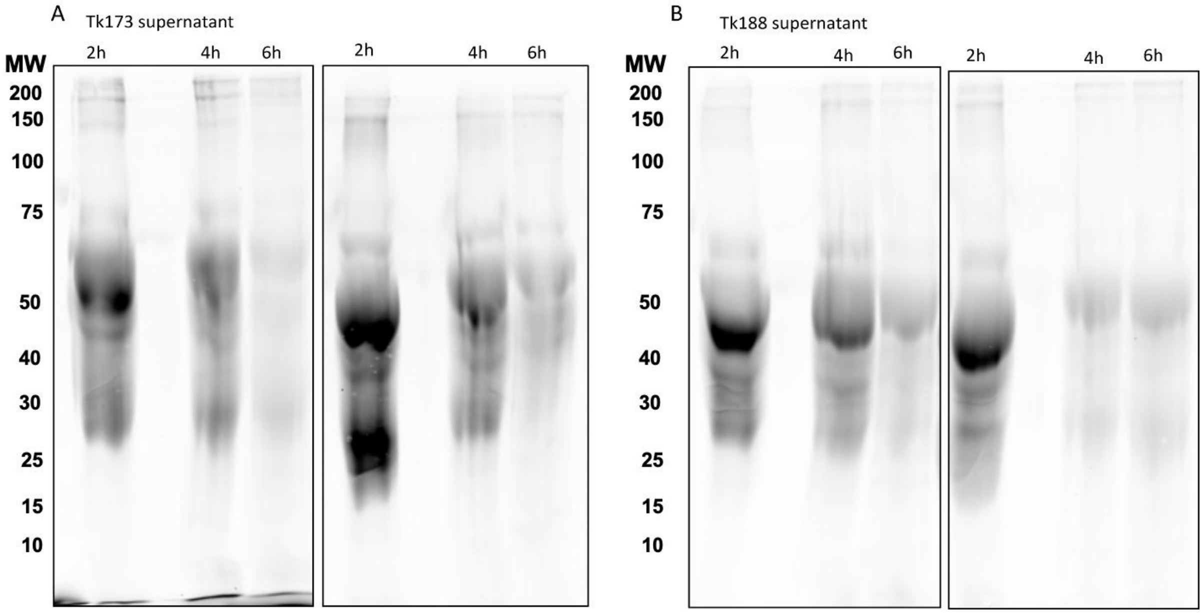

Supplemental Figure 1

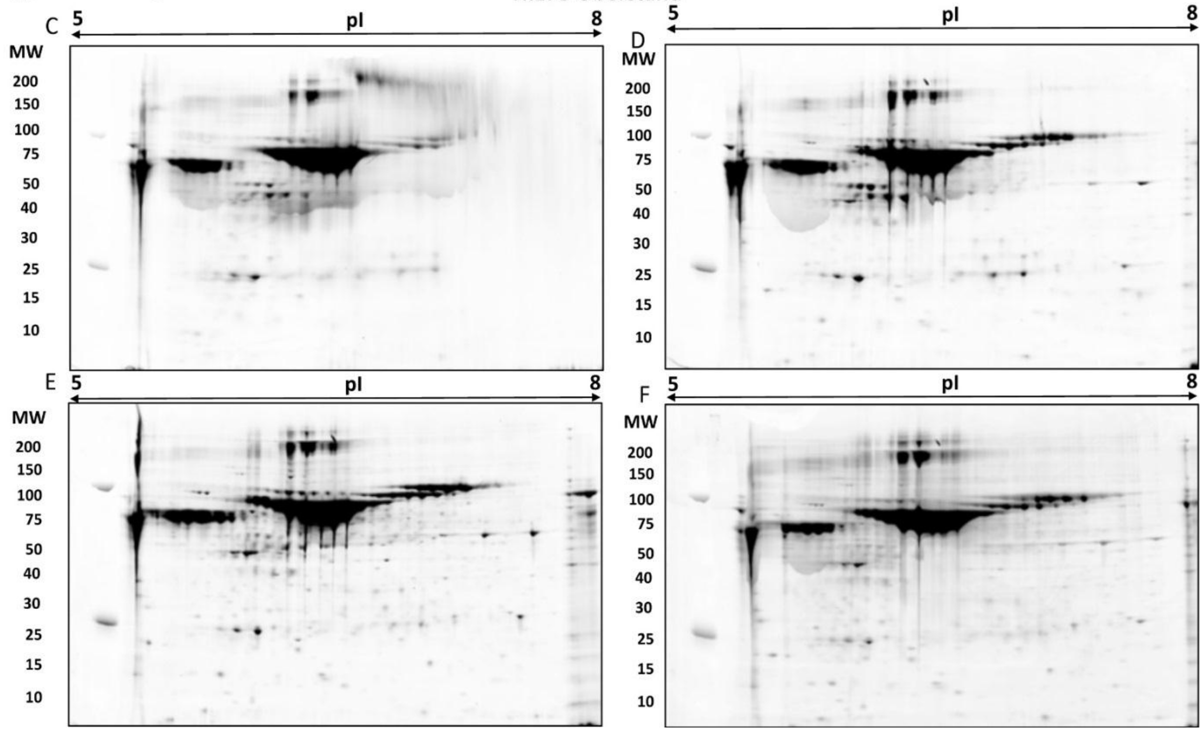

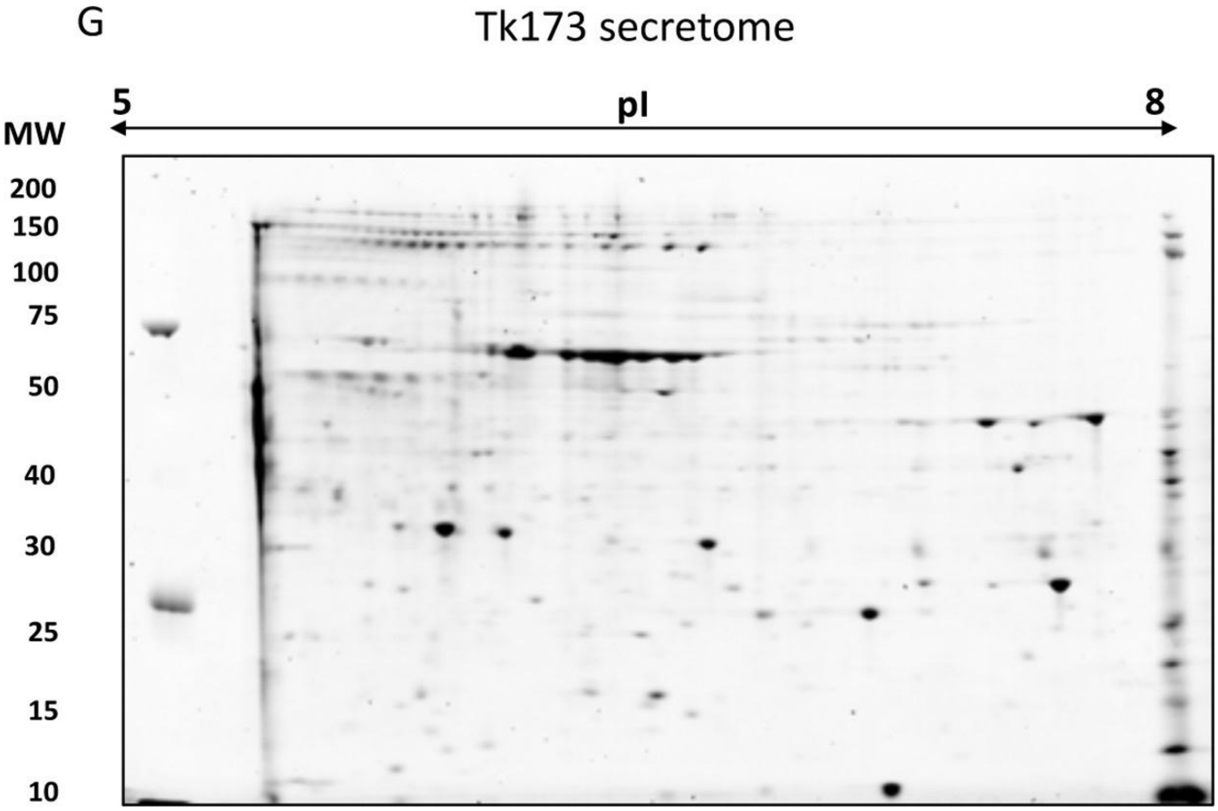

Figure S1. Secretome enrichment: protocol optimization. 1D SDS-PAGE documentation of washing steps: Culture medium was substituted with FCS-free medium, which was changed every 2 h. The supernatants were then collected, and the proteins isolated and separated in 1D SDS-PAGE ((**A**) TK173 and (**B**) TK188). Proteins were stained with Flamingo fluorescent gel stain. Two-dimensional pattern of the proteins isolated from supernatant of TK173, (**C**) 2 h, (**D**) 4 h, (**E**) 6 h, and (**F**) 8 h after changing to FCS-free medium. (G) Cell secretome collected 24 h after elimination of the contaminating FCS-proteins with different washing steps. Proteins were stained with Flamingo fluorescent gel stain.

Supplemental Figure 2

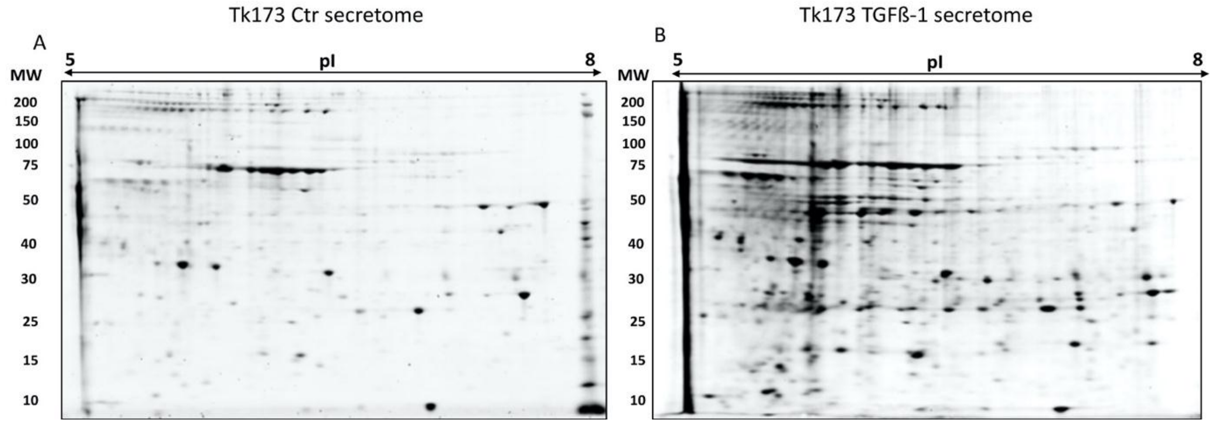

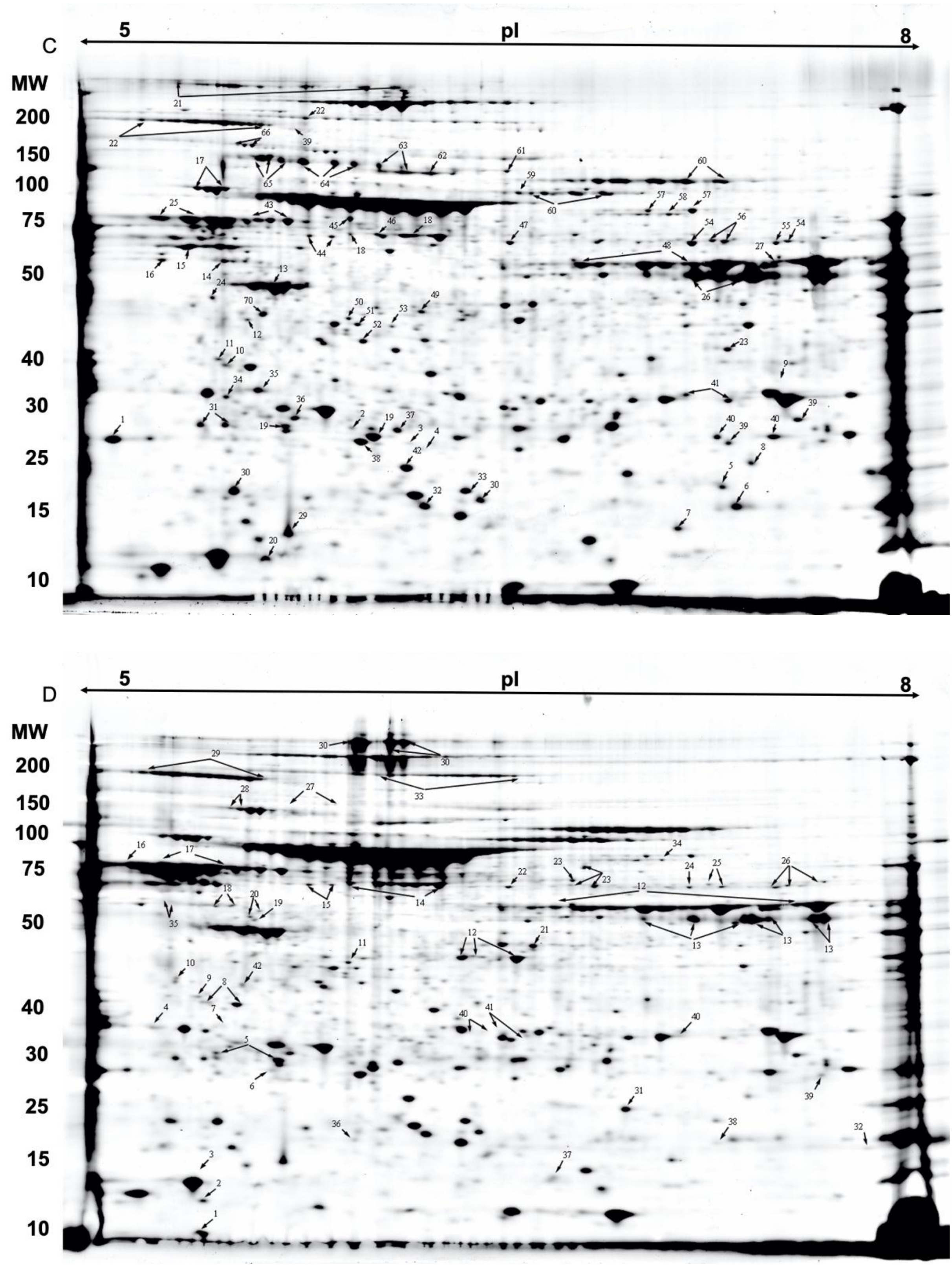

Figure S2. 2-DE reference maps of secretomes; 150 μg proteins were loaded on an 11 cm IPG strip with a linear pH gradient PI 5–8 for IEF; 12% SDS-polyacrylamide gels were used for the second dimension. Proteins were stained with Flamingo fluorescent gel stain. Identified spots were assigned a number corresponding to that in their table. 2-DE maps from secretome of (**A**) TK173 control and (**B**) TGFβ1-treated ones. The 2-DE patterns revealed an alteration of secretome in stimulated TK173. Secretome patterns from TK173 treated with (**C**) ANG II and (**D**) PDGF.

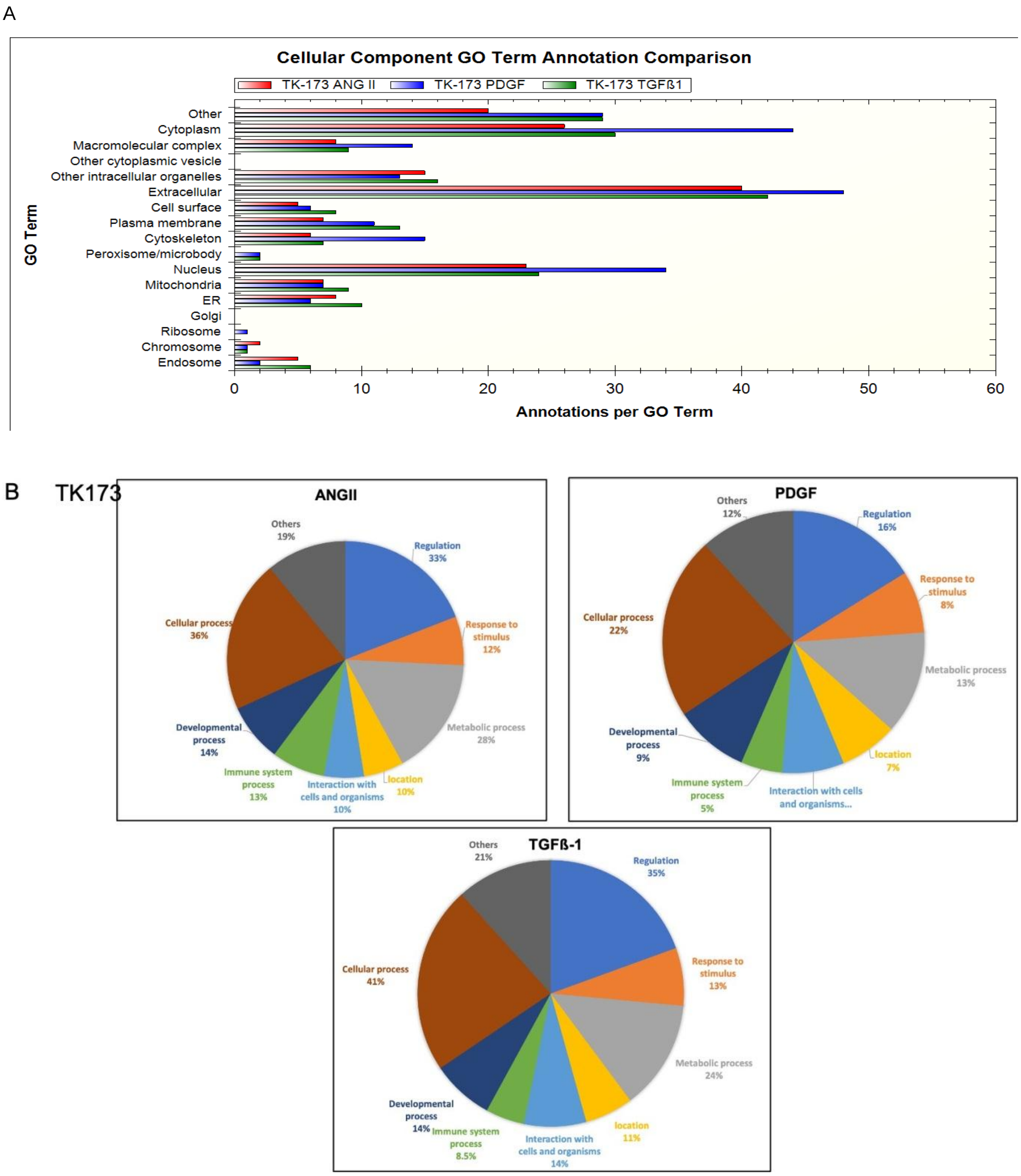

Figure S3. Classification of the differentially expressed proteins upon ANG II, TGFβ1, or PDGF treatment in TK173. (**A**) Bar charts of the cellular component analyzed by STRAP biological function analysis in which the identified proteins from all treatments in both cell types are involved. GO analysis of datasets shows a clear enrichment for extracellular proteins in TK173 secretome. (**B**) Pie charts illustrating the classification of the identified secretome protein according to their biological processes (STRAP GO annotations) and the treatment used.

Supplemental Figure 4

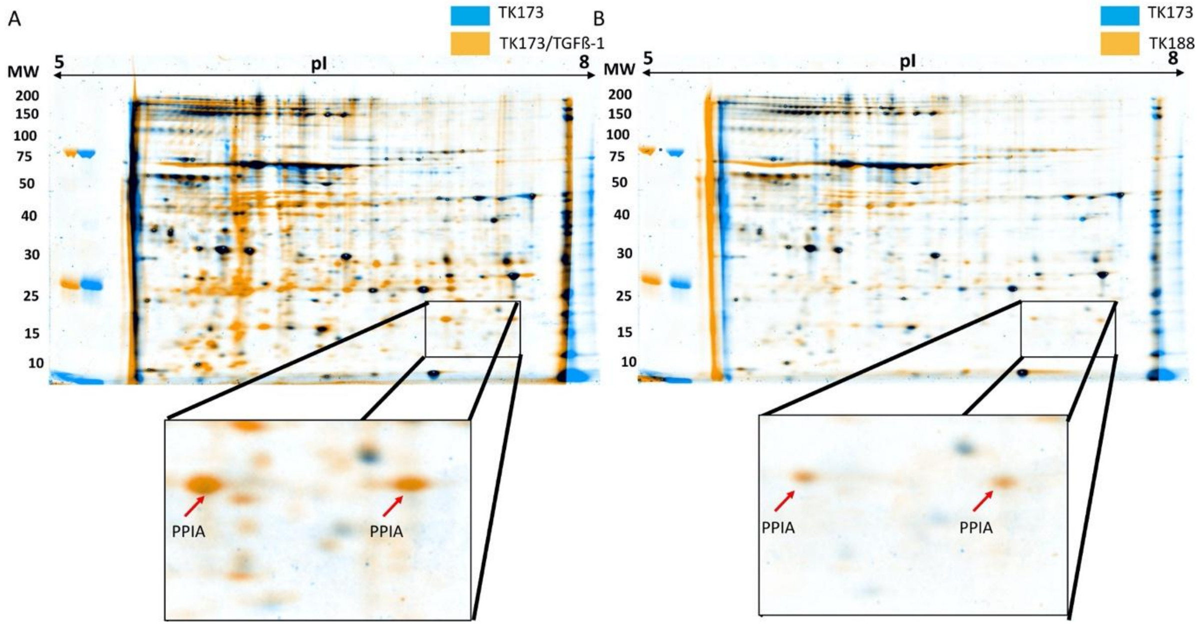

Figure S4. Two-dimensional pattern of total proteins isolated from TK173, TK173/TGFβ1, and TK188 secretomes. (**A**) Overlapping of the 2D maps from TK173 and TK173/TGFβ1 secretomes. (**B**) Overlapping of 2D maps from TK173 and TK188 secretomes. Close-up regions showing the PPIA spots in the secretome from both overlapping. The proteins (150 μg) were loaded and separated by 2-DE according to PI and MW. The protein spots were visualized by Flamingo fluorescent gel stain.

A

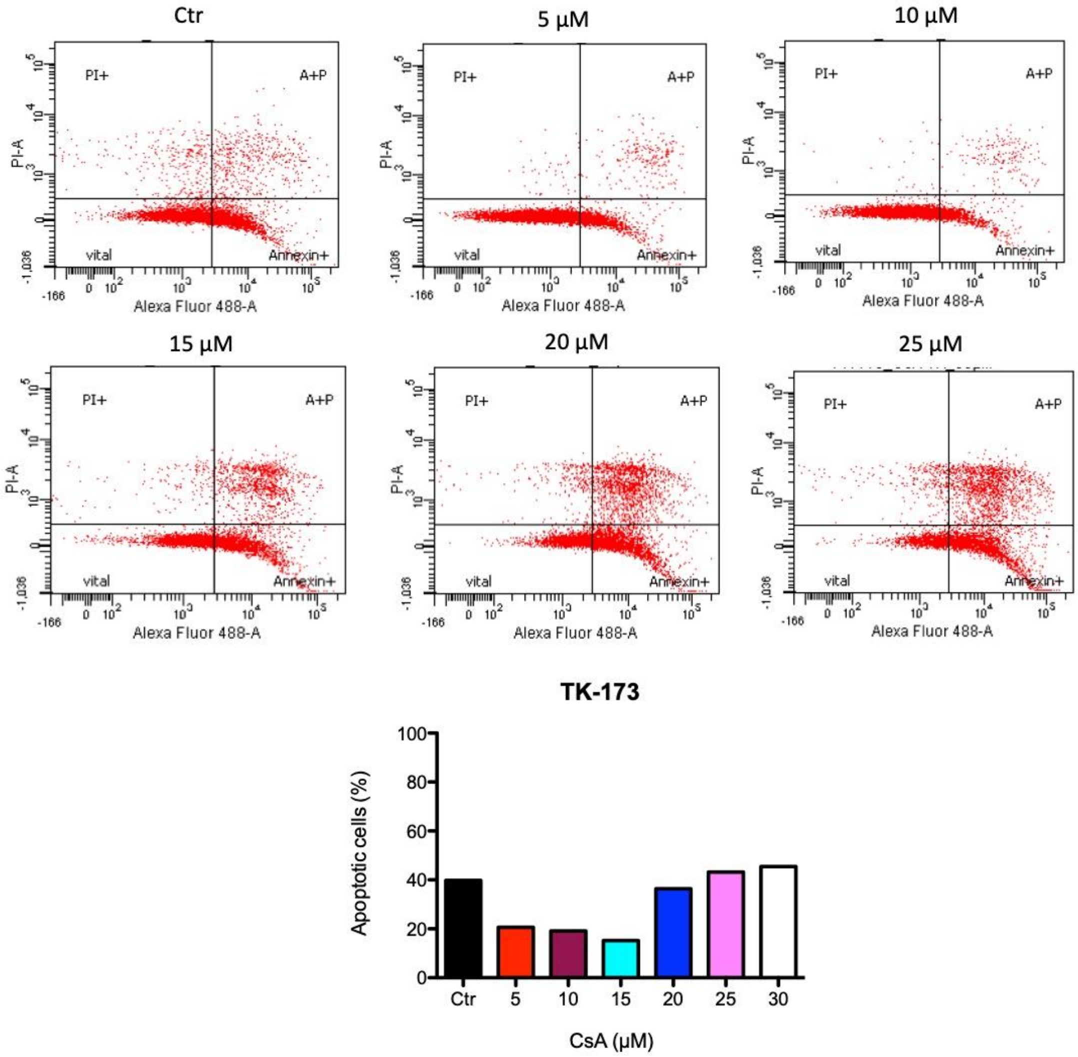
Ctr
5 µM
10 µM
15 µM
20 µM
25 µM
PI+
A+P
vital
Annexin+
PI-A
Alexa Fluor 488-A
TK-173
Apoptotic cells (%)
Ctr
5
10
15
20
25
30
CsA (µM)

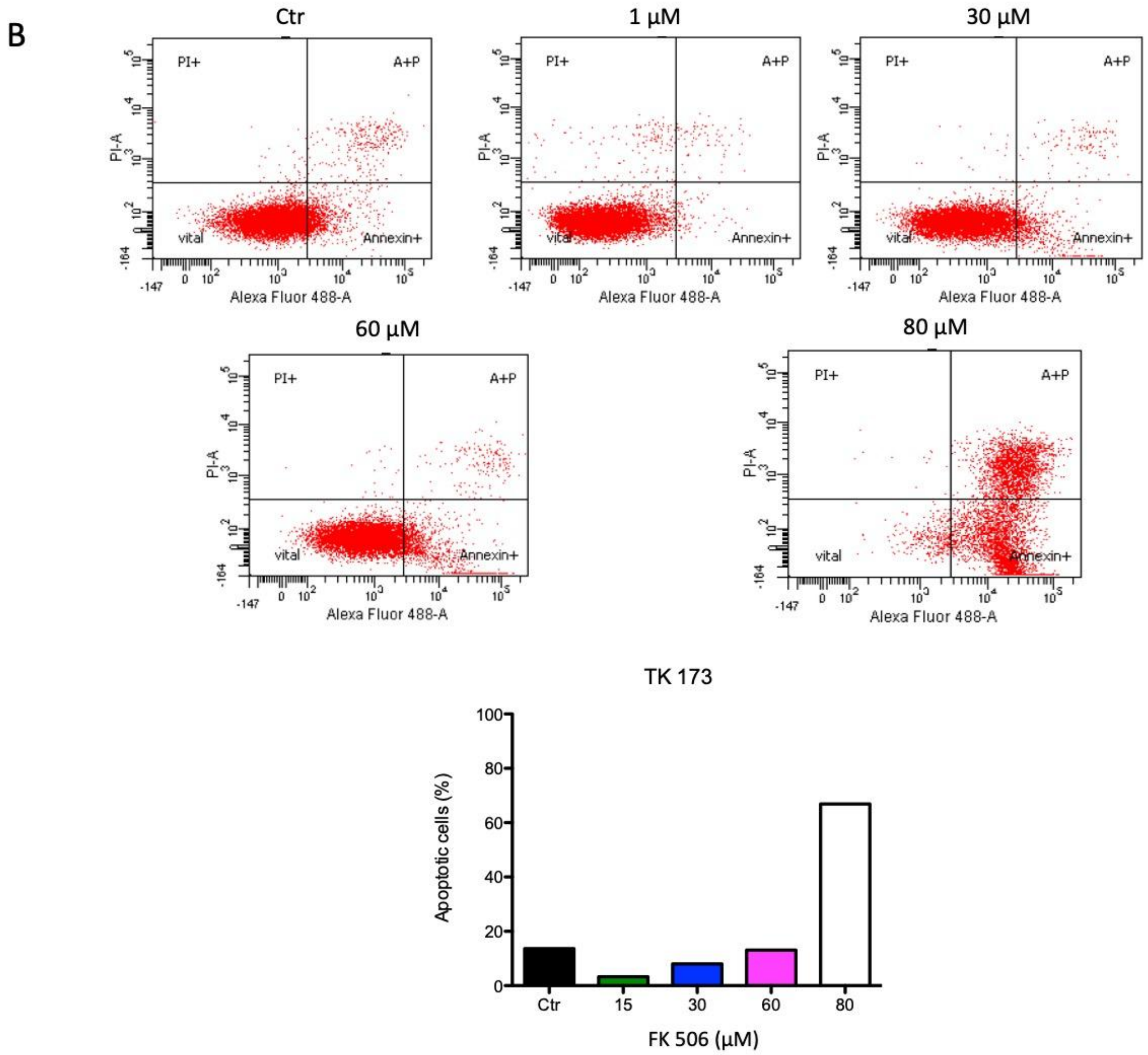

Figure S5. TK173 cells were treated with different doses of (**A**) CsA (5-30 µM) or (**B**) FK506 (15–80 µM) for 72 h and Annexin V/PI staining was performed to examine the apoptotic cells. Percentage of apoptotic cells after treatment with indicated doses of CsA or FK506 are presented in the form of a bar diagram.

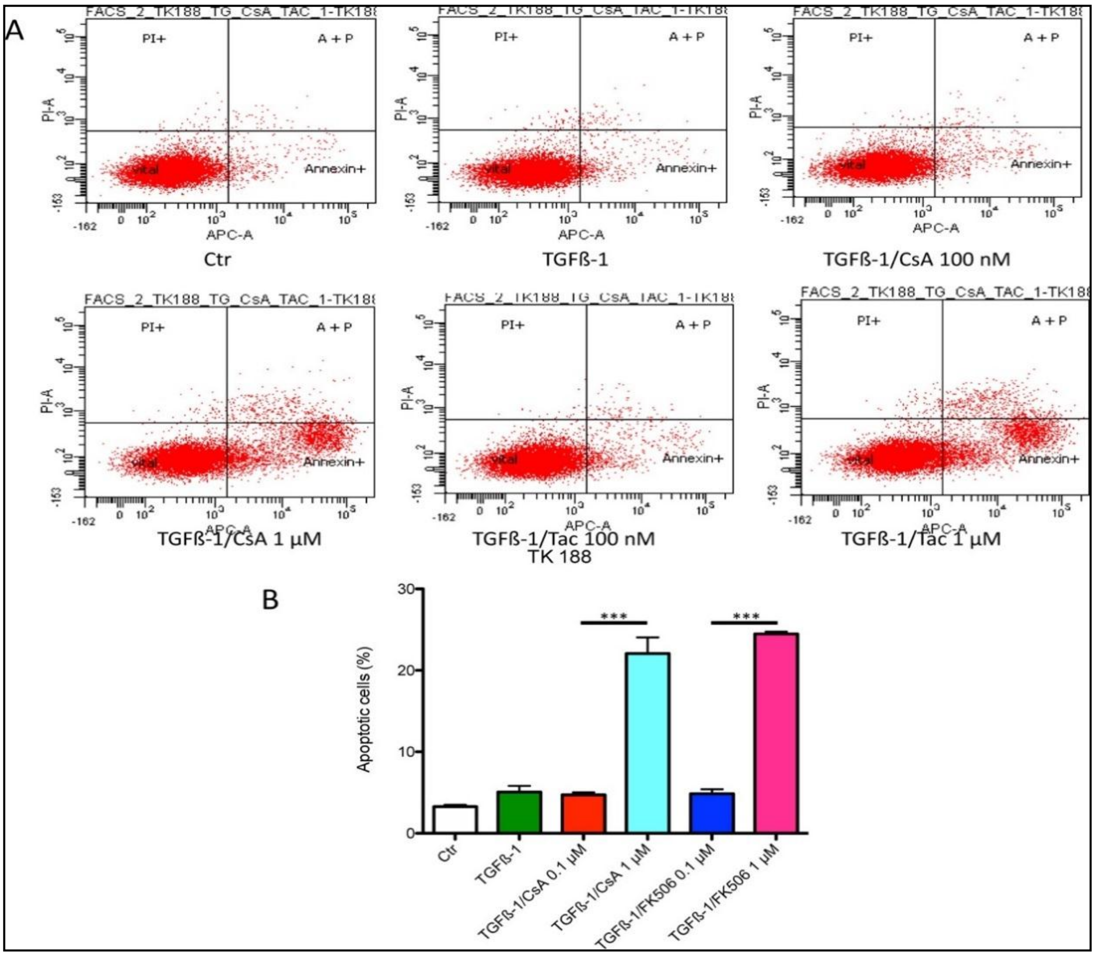
A
FACS_2_TK188_TG_CsA_TAC_1-TK188
PI+
A + P
vital
Annexin+
PI-A
APC-A
Ctr
TGFß-1
TGFß-1/CsA 100 nM
TGFß-1/CsA 1 µM
TGFß-1/Tac 100 nM
TK 188
TGFß-1/Tac 1 µM
B
Apoptotic cells (%)
***
Ctr
TGFß-1
TGFß-1/CsA 0.1 µM
TGFß-1/CsA 1 µM
TGFß-1/FK506 0.1 µM
TGFß-1/FK506 1 µM

C

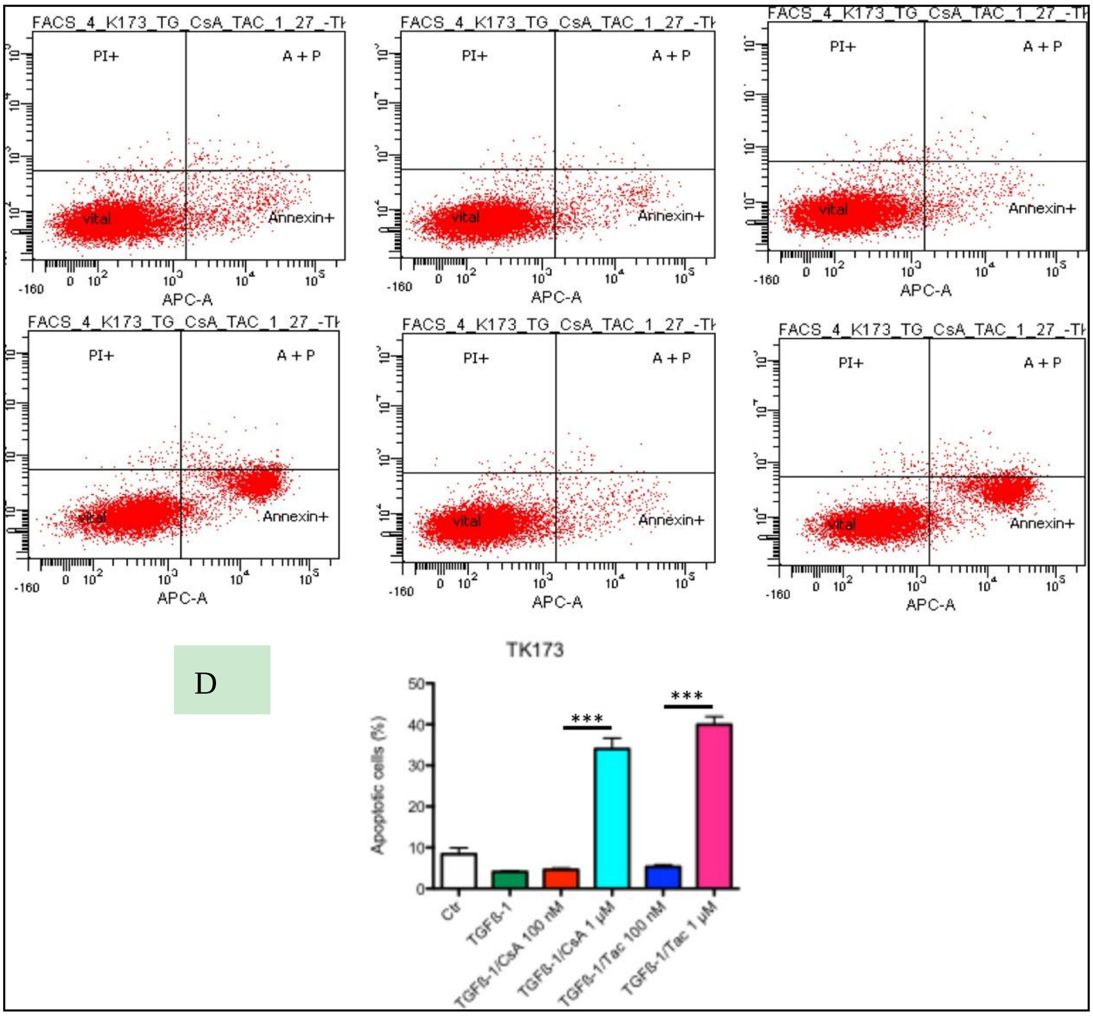

Figure S6. Low concentration of CsA and FK506 did not have significant impact on cell survival under TGFβ1 treatment. TK173 and TK188 cells were treated either with TGFβ1 alone or in combination with PPIases inhibitors (TGFβ1/CsA or TGFβ1/FK506). Two doses of CsA and FK506 (0.1 and 1 μM) were used for 72 h and Annexin V/PI staining was performed to examine the apoptotic cells. The apoptosis assay showed that the percentage of dead cells, under the TGFβ1/CsA or TGFβ1/FK506 combined treatment, depends on the inhibitor concentration. High concentrations of CsA or FK506 (1 μM) significantly increased the number of dead cells for both cell lines TK173 (A, B) and TK188 (C, D). (B, D) Percentage of apoptotic cells after treatment with indicated doses of CsA or FK506. *** p <0.001.

Table S1. List of non-redundant proteins found to be differently secreted in the TGFβ1-treated TK173 cells compared to untreated control. The gene name, accession number in Swiss-Prot, molecular weight, peptide mass fingerprinting (PMF), and MS/MS information are given.

	**Protein name**	**Gene Name**	**Swiss-Prot**	**Mass**	**PMF-Score**	**PMF Sequence Coverage**	**MS/MS Score**	**Peptides**
1	Galectin 1	LGALS1	P09382	14706			153	10
2	Coactosin-like protein	COTL1	Q14019	15935	63	10		
3	Glutathione S-transferase P	GSTP1	P09211	23341	59	10		
4	Retinol-binding protein 4	RBP4	P02753	22995			68	4
5	Ubiquitin carboxyl-terminal hydrolase isozyme L1	UCHL1	P09936	24808	73	14		
6	Rho GDP-dissociation inhibitor 1	ARHGDA	P52565	23193	70	12		
7	Ran-specific GTPase-activating protein	RANBP1	P43487	23296			57	2
8	Proteasome subunit alpha type-3	PSMA3	P25788	28415			115	6
9	Tubulin-folding cofactor B	TBCB	Q99426	27308	87			
10	Chloride intracellular channel protein 1	CLTC1	Q00299	26906	150		106	4
11	Inositol monophophatase 1	IMPA1	P29218	30169			91	3
12	Beta-2-microglobulin	B2M	P61769	13706	67	8	182	8
13	Protein S100-A11	S100A11	P31949	11733			92	7
14	GTP-binding nuclear protein Ran	RAN	P62826	24408	81	11		
15	Peptidyl-prolyl cis-trans isomerase A	PPIA	P62937	18001	216			
16	Heterogenous nuclear ribonucleoproteins A2/B1	HNRNPA2B1	P22626	37407	149			
17	Carbonic anhydrase 1	CA1	P00915	28852	204			
18	S-methyl-5′-thioadenosine phosphorylase	MTAP	Q13126	31216			146	7
19	Glyceraldehyde-3-phosphate dehydrogenase	GAPDH	P04406	36030			96	3
20	Guanine nucleotide-binding protein subunit beta-2-like 1	GNB2L1	P63244	35055	78	13		

21	Proteasome subunit alpha type-1	PSMA1	P25786	29537			123	5
22	Purine nucleoside phosphorylase	NP	P00491	32097	106	14		
23	Aldose reductase	AKR1B1	P15121	35830	120	13		
24	S-formylglutathione hydrolase	ESD	P10768	31442	62	8		
25	Triosephosphate isomerase	TPI1	P60174	26653	256	53		
26	Endoplasmic reticulum protein Erp29	ERP29	P30040	28975			220	6
27	Metalloproteinase inhibitor 2	TIMP2	P16035	24383			44	2
28	Ubiquitin-conjugating enzyme E2 N	UBE2N	P61088	17127	65	10		
29	Ubiquitin-conjugating enzyme E2 N variant2	UBE2V2	Q15819	16352	58	9		
30	78 kDa glucose-regulated protein	HSPA5	P11021	72288	156	32		
31	Inorganic pyrophosphatase	PPA1	Q15181	32639	88	11	48	3
32	F-actin-capping protein subunit alpha-1	CAPZA1	P52907	32902	63	11	131	5
33	Glyoxalase domain-containing protein 4	GLOD4	Q9HC38	34771			87	1
34	Vimentin	VIM	P08670	53619	180	44	972	37
35	Actin, cytoplasmic 1	ACTB	P60709	41710	109	17		
36	Actin, cytoplasmic 2	ACTG1	P63261	41766	74	13	343	8
37	Plasminogen activator inhibitor 1	SERPINE1	P05121	45031	191	35	429	6
38	Alpha-enolase	ENO1	P06733	47139	160	28	396	16
39	Glutathione synthetase	GSS	P48637	52352	133	28		
40	Protein disulfide-isomerase A3	PDIA3	P30101	56747	114	21	454	4
41	Tryptophanyl-tRNA synthetase, cytoplasmic	WARS	P23381	53132			173	8
42	Cytosol aminopeptidase	LAP3	P28838	56131			76	5
43	T-complex protein 1 subunit beta	CCT2	P78371	57452			131	9
44	D-3-phosphoglycerate dehydrogenase	PHGDH	O43175	56614			274	6
45	Inosine-5´monophosphate dehydrogenase 2	IMPDH2	P12268	55770			84	5

46	UDP-glucose 6-dehydrogenase	UGDH	O60701	54989			107	3
47	Transforming growth factor-beta-induced protein ig-h3	TGFBI	Q15582	74634	127	19		
48	Pyruvate kinase isozymes M1/M2	PKM2	P14618	57900	91	17		
49	Stress-induced-phosphoprotein 1	STIP1	P31948	62599	69	19	244	7
50	Transitional endoplasmic reticulum ATPase	VCP	P55072	89266	114	32	53	4
51	**Alpha-fetoprotein**	AFP	P02771	68543	78	10	140	8
52	Stress-70 protein, mitochondrial	HSPA9	P38646	73635			320	12
53	Fibronectin	FN1	P02751	262442	78	33	52	3
54	Collagen alpha-1 (I) chain	COL1A1	P02452	138827			428	21
55	Heat shock 70 kDa protein 4	HSPA4	P34932	94271	86	23	47	4
56	Myosine-9	MYH9	P35579	226392			241	12
57	60 kDa heat shock protein, mitochondrial	HSPD1	P10809	61016	101	20		
58	T-complex protein 1 subunit epsilon	CCT5	P48643	59633			83	4
59	Tubulin beta chain	TUBB	P07437	49639	261	44		
60	Eukaryotic translation initiation factor 3 subunit F	EIF3F	O00303	37540			205	6
61	Cofilin 1	CFL1	P23528	18491			116	3

Table S2. List of non-redundant proteins found to be differently secreted in the ANG II-treated TK173 cells compared to untreated control. The gene name, accession number in Swiss-Prot, molecular weight, peptide mass fingerprinting (PMF), and MS/MS information are given.

	Protein name	Gene Name	Swiss-Prot	Mass	PMF-Score	PMF Sequence Coverage	MS/MS Score	Peptides
1	Translationally-controlled tumor protein 1	TPT1	P13693	19583			352	23
2	Heme-binding protein 1	HEBP1	Q9NRV9	21084			111	7
3	Basment membrane-specific heparan sulfate proteoglycan core protein	HSPG2	P98160	468500			313	13
4	Thioredoxine-dependent peroxide reductase, mitochondrial	PRDX3	P30048	27675			110	4
5	Heterogenous nuclear ribonucleoproteins A2/B1	HNRNPA2B1	P22626	37407			149	12
6	Peptidyl-prolyl cis-trans isomerase A	PPIA	P62937	18001			216	20
7	UPF0556 protein C19 or f10	C19 or f10	Q969H8	18783			135	6
8	Transgelin	TAGLN	Q01995	22516			301	15
9	Carbonic anhydrase 1	CA1	P00915	28852			204	8
10	Chloride intracellular channel protein 1	CLIC1	Q00299	26906			234	13
11	EF-hand domain-containing protein D2	EFHD2	Q96C19	26680			74	12
12	Cathepsin L1	CTSL1	P07711	37540			76	12
13	Actin, cytoplasmic 1	ACTB	P60709	41710			1070	64
14	Plasminogen activator inhibitor 1	SERPINE1	P05121	45031			425	24
15	Tubulin beta-2C chain	TUBB2C	Q8N6N5	49799			841	46
16	Tubulin beta chain	TUBB	P07437	49639			305	20
17	78 kDa glucose-regulated protein	HSPA5	P11021	72288			1038	45
18	Protein disulfide-isomerase A3	PDIA3	P30101	56747			140	28
19	Glutathion S-transferase P	GSTP1	P09211	23341	95	12	327	16
20	Thioredoxin domain-containing protein 17	TXNDC17	Q0BRA2	13932			313	11

21	Fibronectin	FN1	P02751	262442			707	47
22	Collagen alpha-1 (VI) chain	COL6A1	P12109	108462			726	34
23	S-formylglutathione hydrolase	ESD	P10768	31442			157	12
24	Serine-threnine kinase receptor-associated protein	STRAP	Q0Y3F4	38414			53	6
25	Tubulin alpha-1A chain	TUBA1A	Q71U36	50104			341	17
26	Procollagen C-endopeptidase enhancer-1	PCOLCE	Q15113	47942			196	13
27	Calsyntenin-1	CLSTN1	Q94985	109724			374	25
28	Coactosin-like protein	COTL1	Q14019	15935			173	18
29	Eukaryotic translation initiation factor 5A-1	EIF5A	P63241	16821			117	6
30	Lactoylglutathione lyase	GLO1	Q04760	20764	58	10		
31	Stathmin	STMN1	P16949	17292			188	10
32	Elongation factor 1-gamma	EEF1G	P26641	50087			207	8
33	Lamin-B1	LMNB1	P20700	66408	85	17		
34	Proteasome subunit alpha type-3	PSMA3	P25788	28415			74	7
35	3-hydroxyisobutyryl-CoA hydrolase, mitochondrial	HIBCH	Q6NVY1	43454	61	12		
36	Proteaosome subunit beta type-4	PSMB4	P28070	29185	71	9		
37	Peroxiredoxin-2	PRDX2	P32119	21878	90	18		
38	Proteasome subunit alpha type-2	PSMA2	P25787	25899			144	10
39	Peroxiredoxin-1	PRDX1	Q06830	22096			199	15
40	Proteasome subunit beta type-2	PSMB2	P49721	22822			183	10
41	Triosephosphate isomerase	TPI1	P60174	26653			579	17
42	Transgelin-2	TAGLN2	P37802	22377	117	14		
43	60 kDa heat shock protein, mitochondrial	HSPD1	P10809	61016	195	27		
44	FK506-binding protein 4	FKBP4	Q02790	51772	87	12	35	7
45	T-complex protein 1 subunit epsilon	CCT5	P48643	59633			174	17

46	Dihydropyrimidinase-related protein 2	DPYSL2	Q16555	62255	67	14		
47	Tryptophanyl-tRNA synthetase, cytoplasmic	WARS	P23381	53132	98	13		
48	Alpha-enolase	ENO1	P06733	47139	170	22		
49	HLA class I histocompatibility antigen A-24 alpha chain	HLA-A	P01892	40663	105	13		
50	Eukaryotic translation initiation factor 3 subunit I	EIF3I	Q13347	36479	80	11		
51	N(G), N(G)-dimethylarginine dimethylaminohydrolase 1	DDAH1	Q94760	31102			141	9
52	Inorganic pyrophosphatase	PPA1	Q15181	32639	127	17		
53	F-actin-capping protein subunit alpha-2	CAPZA2	P47755	32929	98	12		
54	Fascin	FSCN1	Q16658	54496			375	12
55	Dihydrolipoyl dehydrogenase, mitochondrial	DLD	P09622	54116			209	10
56	Cytosol aminopeptidase	LAP3	P28838	56131			887	30
57	Stress-induced-phosphoprotein 1	STIP1	P31948	62599			369	17
58	Bifunctional purine biosynthesis protein PUR+1	ATIC	P31939	64575			277	12
59	Putative fatty acid-binding protein 5-like protein 3	FABP5L3	A8MUU	11292	59	6		
60	Lactotransferrin	LTF	P02788	78132			71	3
61	Gelsolin	GSN	P06396	85644	86	11		
62	Filamin-B	FLNB	O75369	278021			58	3
63	Filamin-A	FLNA	P21333	280564	107	28		
64	Complement C1r subcomponent	C1R	P00736	80067	69	14		
65	Transitional endoplasmic reticulum ATPase	VCP	P55072	89266	98	23	191	21
66	Heat shock 70 kDa protein 4	HSPA4	P34932	94271	111	19	187	17

Table S3. List of non-redundant proteins found to be differently secreted in the PDGF-treated TK173 cells compared to untreated control. The gene name, accession number in Swiss-Prot, molecular weight, peptide mass fingerprinting (PMF), and MS/MS information are given.

Spot nr.	Protein name	Gene Name	Swiss-Prot	Mass	PMF Score	PMF Sequence Coverage	MS/MS Score	Peptides
1	Protein S100-A6	S100A6	P06703	10173			105	4
2	Myotrophin	MTPN	P58546	12887			59	1
3	Coactosin-like protein	COTL1	Q14019	15935	71	11	173	
4	Rho GDP-dissociation inhibitor 1	ARHGDIA	P52565	23193	94	11		
5	Glutathione S-transferase P	GSTP1	P09211	23341	63	7	281	8
6	Peroxiredoxin-2	PRDX2	P32119	21878	90		46	2
7	Proteasome subunit alpha type-3	PSMA3	P25788	28415			44	2
8	Chloride intracellular channel protein 1	CLIC1	Q00299	26906	76	9	189	5
9	Tubulin-folding cofactor B	TBCB	Q99426	27308			87	3
10	Coatomer subunit epsilon	COPE	O14579	34460	69	11		
11	Eukaryotic translation initiation factor 3 subunit I	EIF3I	Q13347	36479	80		93	4
12	Alpha-enolase	ENO1	P06733	47139	140	25		
13	Plasminogen activator inhibitor 1	SERPINE1	P05121	45031	200	21	513	19
14	Protein disulfide-isomerase A3	PDIA3	P30101	56747	110	22	147	3
15	FK 506-binding protein 4	FKBP4	Q02790	51772			44	3
16	Alpha-1-antitrypsin	**SERPINA1**	P01009	46737			519	18
17	Nucleobindin-1	NUCB1	Q02818	53846	105	20		
18	26S protease regulatory subunit 6B	PSMC4	P43686	47337			65	5
19	Actin, cytoplasmic 1	ACTB	P60709	41710			116	6
20	Eukaryotic translation initiation factor 3 subunit F	EIF3F	O00303	37540			56	4

21	Elongation factor 2	EEF2	P13639	95277			54	3
22	Tryptophanyl-tRNA synthetase, cytoplasmic	WARS	P23381	53132	174	18		
23	Pre-mRNA-processing factor 19	PRPF19	Q9UMS4	55146			112	4
24	Cytosol aminopeptidase	LAP3	P28838	56131			291	12
25	Inosine-5′monophosphate dehydrogenase 2	IMPDH2	P12268	55770			89	3
26	Dihydrolipoyl dehydrogenase, mitochondrial	DLD	P09622	54116			193	5
27	Myosin-9	MYH9	P35579	226392			206	12
28	Transitional endoplasmic reticulum ATPase	VCP	P55072	89266	145	32		
29	Collagen alpha-1 (VI) chain	COL6A1	P12109	108462	60	15	247	9
30	Fibronectin	FN1	P02751	262442			574	
31	Cofilin 1	CFL1	P23528	18491			149	5
32	Peptidyl-prolyl cis-trans isomerase A	PPIA	P62937	18001	96	10	186	10
33	Collagen-alpha-2 (VI) chain	COL6A2	P12110	108512	116	23	103	4
34	Stress-induced-phosphoprotein 1	STIP1	P31948	62599			68	3
35	ATP synthase subunit beta, mitochondrial	ATP5B	P06576	56525			171	7
36	Nucleoside diphosphate kinase A	NME1	P15531	17138	86	11		
37	Profilin-1	PFN1	P07737	15045			156	4
38	Lysozyme C	LYZ	P61626	16526	61	10		
39	Phosphatidylethanolamine-binding protein-1	PEBP1	P30086	21044			136	4
40	Triosephosphate isomerase	TPI1	P60174	26653	94	24	579	
41	Enoyl-CoA hydratase, mitochondrial	ECHS1	P30084	31367			117	3

Table S4. List of potential interaction partner of PPIA identified and quantified using mass spectrometry. IEP: isoelectric point; mw: molecular weight; CTR: control; TGF: TGFβ1 treated.

description	Gene name	accession	entry	IEP	mw	CTR	TGF
Peptidyl-prolyl cis-trans isomerase A	PPIA	P62937	PPIA_HUMAI	7,85	18240,64	411,95	568,08
Heterogeneous nuclear ribonucleoprotein U	HNRNPU	Q00839	HNRPU_HUN	5,66	91325,94		329,21
Ubiquitin-40S ribosomal protein S27a	RPS27A	P62979	RS27A_HUM	10,24	18307,10		135,32
Galectin-1	LGALS1	P09382	LEG1_HUMA	5,15	15057,89	61,14	103,95
E3 SUMO-protein ligase RanBP2	RANBP2	P49792	RBP2_HUMA	5,81	362590,78		78,65
Heat shock protein 75 kDa_ mitochondrial	TRAP1	Q12931	TRAP1_HUM	8,31	80395,28	11,33	69,58
Fibronectin	FN1	P02751	FINC_HUMA	5,36	266217,54		64,91
Plasminogen activator inhibitor 1	SERPINE1	P05121	PAI1_HUMAI	6,79	45117,13	5,66	56,30
Bifunctional purine biosynthesis protein PURH	ATIC	P31939	PUR9_HUMA	6,30	65129,21	10,66	43,70
Intercellular adhesion molecule 1	ICAM1	P05362	ICAM1_HUM	7,92	58623,66	18,39	40,33
Splicing factor 3B subunit 1	SF3B1	O75533	SF3B1_HUM	6,69	146572,02	2,43	36,54
Myosin-10	MYH10	P35580	MYH10_HUN	5,28	229968,92	7,87	31,49
Peptidyl-prolyl cis-trans isomerase B	PPIB	P23284	PPIB_HUMAI	10,06	23799,61	14,31	29,33
Ras-related protein Rap-1A	RAP1A	P62834	RAP1A_HUM	6,53	21329,38		28,94
40S ribosomal protein S20	RPS20	P60866	RS20_HUMA	10,71	13486,79		27,24
Aldo-keto reductase family 1 member B1	AKR1B1	P15121	ALDR_HUMA	6,59	36252,64	8,73	27,09
Brain acid soluble protein 1	BASP1	P80723	BASP1_HUM	4,42	22693,42	6,91	21,55
Myosin-11	MYH11	P35749	MYH11_HUN	5,25	228195,03	4,50	21,10
Ras-related C3 botulinum toxin substrate 1	RAC1	P63000	RAC1_HUMA	8,58	21849,32		20,56
Tumor protein D54	TPD52L2	O43399	TPD54_HUM	5,08	22294,78	4,45	13,04
Integrin alpha-5	ITGA5	P08648	ITA5_HUMAI	5,41	115677,14	4,45	11,68
Cytoplasmic dynein 1 heavy chain 1	DYNC1H1	Q14204	DYHC1_HUM	5,99	535145,98		10,62
Bifunctional glutamate/proline--tRNA ligase	EPRS	P07814	SYEP_HUMA	7,00	172187,91		9,30
CD166 antigen	ALCAM	Q13740	CD166_HUM	5,87	65786,70	4,70	9,04
Serpin B12	SERPINB12	Q96P63	SPB12_HUM	5,22	46675,69		8,14
Small nuclear ribonucleoprotein F	SNRPF	P62306	RUXF_HUMA	4,43	9782,26		8,03
Integrin alpha-V OS=Homo sapiens	ITGAV	P06756	ITAV_HUMA	5,32	117121,56		7,89
Thrombospondin-1	THBS1	P07996	TSP1_HUMA	4,53	133374,73		7,83
Proteasome subunit beta type-1	PSMB1	P20618	PSB1_HUMA	8,22	26717,54		7,69
Protein S100-A10	S100A10	P60903	S10AA_HUM	7,27	11317,21		7,46
S-phase kinase-associated protein 1	SKP1	P63208	SKP1_HUMA	4,20	18829,12		7,42
Splicing factor_ proline- and glutamine-rich	SEPQ	P23246	SFPQ_HUMA	9,95	76263,60		7,39
LIM and SH3 domain protein 1	LASP1	Q14847	LASP1_HUM.	6,70	30116,41		7,30
Coatomer subunit beta	COPB1	P53618	COPB_HUMA	5,66	108282,98		6,84
Isoleucine--tRNA ligase_ cytoplasmic	IARS	P41252	SYIC_HUMAI	5,77	145809,96		6,35
Heterogeneous nuclear ribonucleoprotein D-like	HNRNPDL	O14979	HNRDL_HUN	9,96	46608,67		6,26
Tropomyosin beta chain	TPM2	P07951	TPM2_HUMA	4,46	32964,85		5,84
GTPase NRas	NRAS	P01111	RASN_HUMA	4,82	21514,35		5,82
Cystatin-A	CSTA	P01040	CYTA_HUMA	5,22	11006,51		5,36
Protein Niban	FAM129A	Q9BZQ8	NIBAN_HUM	4,54	104104,30		5,10
ELAV-like protein 1	ELAVL1	Q15717	ELAV1_HUM	9,57	36263,04		5,07
Podocalyxin	PODXL	O00592	PODXL_HUM	5,16	59091,56		4,99
Putative heat shock protein HSP 90-beta 2	HSP90AB2P	Q58FF8	H90B2_HUM	4,59	44520,09		4,97
Ephrin type-A receptor 2	EPHA2	P29317	EPHA2_HUM	5,83	109749,33		4,64
Collagen alpha-1(VI) chain	COL6A1	P12109	CO6A1_HUM	5,09	109670,06		4,58
Transportin-1	TNPO1	Q92973	TNPO1_HUM	4,65	103837,95		4,46
Phosphatidylinositol transfer protein beta isoform	PITPNB	P48739	PIPNB_HUM.	6,47	31825,26		4,46
Thioredoxin-dependent peroxide reductase_ mitochondrial	PRDX3	P30048	PRDX3_HUM	7,70	28034,84		4,13
Putative heat shock protein HSP 90-beta 4	HSP90AB4P	Q58FF6	H90B4_HUM	4,45	58891,78		4,00
Phosphoribosylformylglycinamidine synthase	PFAS	O15067	PUR4_HUMA	5,41	146388,37		3,94
Epidermal growth factor receptor	EGFR	P00533	EGFR_HUMA	6,27	137699,25		3,85
Glucosidase 2 subunit beta	PRKCSH	P14314	GLU2B_HUM	4,13	60394,97		3,83
Peripherin	PRPH	P41219	PERI_HUMAI	5,21	53765,00		3,74
Ras GTPase-activating-like protein IQGAP3	IQGAP3	Q86VI3	IQGA3_HUM	7,37	185383,40		3,64
Hippocalcin-like protein 1	HPCAL1	P37235	HPCL1_HUM.	5,03	22427,29		3,54
Glial fibrillary acidic protein	GFAP	P14136	GFAP_HUMA	5,26	49937,33		3,41
Ras-related protein Ral-B	RALB	P11234	RALB_HUMA	6,26	23522,64		3,38
Structural maintenance of chromosomes protein 4	SMC4	Q9NTJ3	SMC4_HUMA	6,38	147866,56		3,34
Aminoacyl tRNA synthase complex-interacting multifunctional protein 2	AIMP2	Q13155	AIMP2_HUM	8,27	35691,02		3,31
Ras suppressor protein 1	RSU1	Q15404	RSU1_HUMA	9,23	31540,35		3,31
Ras-related protein Rab-23	RAB23	Q9ULC3	RAB23_HUM	6,24	26887,39		3,21
Collagen alpha-2(VI) chain	COL6A2	P12110	CO6A2_HUM	5,81	109777,13		2,90
Structural maintenance of chromosomes protein 2	SMC2	O95347	SMC2_HUMA	8,73	136169,56		2,75
FACT complex subunit SPT16	SUPT16H	Q9Y5B9	SP16H_HUM	5,37	120484,25		2,71
Aminopeptidase N	ANPEP	P15144	AMPN_HUM	5,16	109938,95		2,62
Glycogen phosphorylase_ brain form	PYGB	P11216	PYGB_HUMA	6,43	97380,45		2,54
CAD protein	CAD	P27708	PYR1_HUMA	6,03	245322,25		2,49
DNA replication licensing factor MCM2	MCM2	P49736	MCM2_HUM	5,20	102580,63		2,45
Signal transducer and activator of transcription 2	STAT2	P52630	STAT2_HUM	5,20	98657,73		2,44
Valine--tRNA ligase	VARS	P26640	SYVC_HUMA	7,37	141730,79		2,30
Peroxidasin homolog	PXDN	Q92626	PXDN_HUMA	6,79	167898,08		2,23
Microtubule-associated protein 4	MAP4	P27816	MAP4_HUM.	5,14	121518,45		2,15
Myoferlin	MYOF	Q9NZM1	MYOF_HUM.	5,79	236248,81		2,13
Kinesin-like protein KIF23	KIF23	Q02241	KIF23_HUMA	8,59	111085,51		2,03
Procollagen-lysine_2-oxoglutarate 5-dioxygenase 2	PLOD2	O00469	PLOD2_HUM	6,27	85427,10		1,45
